# From Oxidized PrNi_0.9_Al_0.1_O_3_ to Reduced PrNi_0.9_Al_0.1_O_2+δ_ Perovskite Nickelates: Stabilization of Infinite-Layer Specimens with Monovalent Ni in the Bulk Polycrystalline Form

**DOI:** 10.1021/acs.inorgchem.5c02051

**Published:** 2025-07-22

**Authors:** Javier Gainza, Carlos A. López, Romualdo S. Silva Jr, João Elias F. S. Rodrigues, Federico Serrano-Sánchez, Alina Skorynina, Norbert M. Nemes, María T. Fernández-Díaz, José Luis Martínez, José Antonio Alonso

**Affiliations:** † Instituto de Ciencia de Materiales de Madrid, CSIC, Cantoblanco, 28049 Madrid, Spain; ‡ European Synchrotron Radiation Facility (ESRF), 71 Avenue des Martyrs, 38000 Grenoble, France; § INTEQUI, (UNSL-CONICET) and Facultad de Química, Bioquímica y Farmacia, UNSL, Almirante Brown 1455, 5700 San Luis, Argentina; ∥ Departamento de Física de Materiales, 16734 Universidad Complutense de Madrid, E-28040 Madrid, Spain; ⊥ CELLS-ALBA Synchrotron Light Source, 08290 Barcelona E, Spain; # 56053 Institut Laue Langevin, 38000 Cedex Grenoble, France

## Abstract

Recently, a new class of high-temperature superconductors, *R*NiO_2_ (where *R* represents rare-earth elements) with infinite-layer (IL) structure, has been identified. They possess the same structural framework as the renowned high-*T*_c_ cuprates but with nickel replacing copper as the central element. In this study, we successfully synthesized infinite-layer samples of PrNi_0.9_Al_0.1_O_2+δ_ in the bulk polycrystalline form through topotactic reduction of the PrNi_0.9_Al_0.1_O_3_ orthorhombic perovskite, via treatment with CaH_2_. The incorporation of aluminum at the octahedral sites promotes the stabilization of bulk derivatives of the infinite-layer structure since unreduced [AlO_6_] octahedra keep the layers together and prevent their decomposition. The lack of superconductivity in bulk samples has been a subject of intense debate in recent literature. One major theoretical question concerns whether hydrogen becomes incorporated into the structure during the reduction from RNiO_3_ to RNiO_2_—as suggested by theory. Here, we present neutron powder diffraction data demonstrating that hydride ions indeed reside within the IL lattice in samples of stoichiometry PrNi_0.9_Al_0.1_O_2.10_H_0.16_. Additional crystallographic analyses were carried out using temperature-dependent synchrotron X-ray diffraction on both reduced and oxidized phases. Furthermore, spectroscopic analysis via XAS and magnetometry confirms the reduction of Ni^3+^ to the Ni^+^ oxidation state, aligning with the crystallochemical evidence.

## Graphical abstract

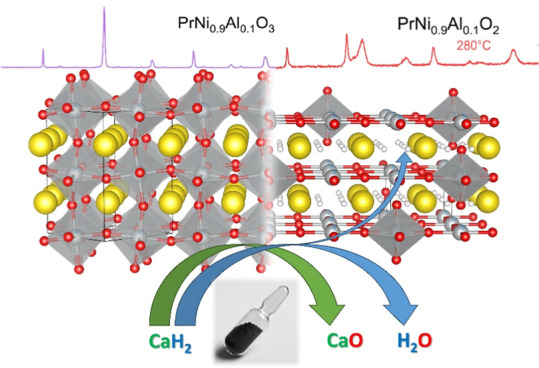

## 1 Introduction

A few years ago, the field of high-temperature superconductivity received a long-awaited breakthrough due to the discovery of this phenomenon in infinite-layer (IL) nickelates, a family of materials analogous to the widely known cuprates. IL nickelates share important similarities with superconducting cuprates, such as comparable crystalline structures and nominal *d* ^9^ electronic configuration of the transition metal, either Ni^+^ or Cu^2+^. The mentioned structural arrangement consists of planes of square-planar [NiO_4_] units connected at their vertices and separated from each other by rare-earth-type atoms (*R*). The first discovery of superconductivity in nickelates was reported by Li et al.^1^ in Nd_0.8_Sr_0.2_NiO_2_ thin films, being reproduced by other groups soon after.^2,3^ To date, superconductivity has also been confirmed in thin films of La-,^2,4^ Pr- ^3,5,6^ and Nd-,^7^ as well as other layered nickel compositions such as Nd_6_Ni_5_O_12_.^8^

Since the initial discovery of superconductivity in nickelates was limited to thin films, significant efforts have been dedicated to replicating these findings in different bulk rare-earth nickelates.^9–12^ However, it is known that the synthesis of nickelates presents several difficulties, so achieving high-quality IL nickelate oxides is already a success. The *R*NiO_3_ precursor itself requires a nickel oxidation state of +3, something only achievable by synthesis at high-temperature and high-oxygen pressure. On the other hand, obtaining the infinite-layer phase requires applying a chemical reduction process to change the Ni-oxidation state from Ni^3+^ to Ni^+^, a quite uncommon oxidation state for Ni. This reduction process has a very narrow temperature window to succeed, which adds to the possibility that the material may decompose during the process. All of this together explains the limited number of reports on nickelates even in the bulk form. Nevertheless, the temperature at which the nickelate is reduced seems to play a role in determining the superconducting *T*_c_ of the infinite-layer and the corresponding superconducting dome, as has been reported for the La_1–*x*_Sr_*x*_NiO_2_ thin-film composition.^13^

One parameter that may be key in some systems for the emergence of superconducting properties is the effect of high pressure.^14–16^ Sun et al.^17^ were pioneers discovering some signatures of superconductivity in the La_3_Ni_2_O_7_ Ruddelsden-Popper phase, with maximum *T*_c_ as high as ∼80 K at pressures between 14 and 43.5 GPa. A recent report also suggests that these signatures can also be observed in thin-film La_3_Ni_2_O_7_ at room pressure, applying epitaxial compressive strain instead.^18^ However, in either case, these results were achieved in single crystals or thin films, not in bulk polycrystalline nickelates. It was not until 2023 that some reports of superconductivity in bulk nickelates began to appear in the literature. First, there was the report of zero resistivity at 9 GPa in polycrystalline La_3_Ni_2_O_7−δ_.^19^ Shortly after, and published by the same group, we received the confirmation of superconductivity with observed zero resistivity and diamagnetism in the La_2_PrNi_2_O_7_ compound.^20^ It is therefore a time when attention is also paid to nickelates in the bulk form, so any extra information about these compositions can be helpful to shed new light in this area.

We have studied different bulk polycrystalline nickelates in the past, characterizing in detail the crystalline structure of, for instance, PrNiO_3_,^21,22^ NdNiO_3_,^23^ SmNiO_3_,^24^ TlNiO_3_,^25^ and LuNiO_3_.^26^ Recently, we have also worked on LaNi_1–*y*_Al_*y*_O_3_ and its infinite-layer counterpart LaNi_0.9_Al_0.1_O_2_,^12^ finding that the infinite-layer phase can be stabilized more easily by substituting part of the Ni with Al. We found signatures of the presence of interlayer hydrogen, still posing an open question as to being necessary for the existence of superconductivity in these compounds.^27–29^ In the present report, we describe the reduction of the closely related orthorhombic PrNi_1–*y*_Al_*y*_O_3_ perovskite and its infinite-layer tetragonal phase, PrNi_1–*y*_Al_*y*_O_2+δ_. In addition, in this case, we found evidence from neutron powder diffraction (NPD) data on the presence of hydrogen in interstitial positions in samples with a stoichiometry of PrNi_0.9_Al_0.1_O_2.10_H_0.16_. This is perhaps the reason for the absence of superconductivity in these bulk compounds. The crystallographic scrutiny, together with X-ray absorption techniques, confirms the presence of monovalent nickel in these reduced IL samples.

## 2 Experimental Methods

### 2.1 Synthesis

Polycrystalline PrNi_1–*y*_Al_*y*_O_3_ nickelates (*y* = 0, 0.1) were synthesized through a citrate-nitrate method. Pr_6_O_11_ (∼99.9%, REO, Alfa-Aesar), Al­(NO_3_)_3_·9H_2_O (>98.5%, Merck), and Ni­(NO_3_)_2_·6H_2_O (≥98.5%, Sigma-Aldrich) were dissolved in citric acid, with nitric acid added to aid the dissolution process. The resulting solution was heated on a hot plate to remove water, forming a viscous organic resin. Such a resin was subsequently dried and thermally decomposed by gradually heating to 600 °C in air over 12 h. The resulting material was then calcined at 800 °C in air for 2 h, yielding a reactive precursor powder. The precursors were further treated at 900 °C under 200 bar of oxygen for 12 h in a high-pressure Morris Research furnace. Finally, the samples were cooled at a rate of 2 °C/min down to room temperature, resulting in polycrystalline-oxidized PrNi_1–*y*_Al_*y*_O_3_ nickelates.

For the chemical reduction process, we used a procedure like that reported by Hayward et al. in the past,^30,31^ which has also proven successful in synthesizing the LaNi_0.9_Al_0.1_O_2_ composition.^12^ The prepared powder samples were mixed with CaH_2_ in a 3:1 weight ratio, thoroughly ground, and placed in Pyrex tubes. These tubes were vacuum-sealed and heated in a furnace. The heating process involved raising the temperature to various levels (180, 250, 280, and 300 °C) in 30 min, maintaining the target temperature for 6 h, and then allowing the samples to cool to room temperature. Before breaking the Pyrex tube, the presence of metallic Ni could be roughly assessed by holding a magnet near the powder to check for the magnetic response, providing a preliminary estimate of the Ni content. Once the tube was opened, the powder was treated with a 0.1 M NH_4_Cl solution in methanol (CH_3_OH) for removing any CaO formed during the reduction. The mixture was left to react at room temperature for 1–2 h, after which it was filtered to isolate the infinite-layer nickelate in powder form.

### 2.2 Powder X-ray and Neutron Diffraction

The structural analysis of both oxidized and reduced powders was conducted by using laboratory X-ray diffraction on a Bruker D8 Advance instrument with Cu-*K*α radiation. High-resolution synchrotron X-ray diffraction (SXRD) data were also collected at the ESRF (European Synchrotron Radiation Facility, Grenoble) on the ID22 beamline. The measurements were performed at a wavelength of λ = 0.35439 Å (35 keV), using 0.5 mm glass or quartz sealed capillaries, continuously rotated to minimize texture effects. The choice of 35 keV X-ray energy helped reduce absorption. Data were recorded over a 2θ range of 1–40° in the continuous scan mode. SXRD data processing followed the method outlined by Fitch and Dejoie.^32^

NPD was also employed for selected samples using the D2B high-resolution diffractometer at the Institut Laue Langevin (ILL) in Grenoble. The NPD measurements were performed with a wavelength of λ = 1.594 Å. Samples with nominal compositions PrNi_0.9_Al_0.1_O_3_ (oxidized precursor) and PrNi_0.9_Al_0.1_O_2+δ_ (reduced at 300 °C) were analyzed at 298 K. Additionally, a PrNi_0.9_Al_0.1_O_3_ sample treated at 180 °C (i.e., just below the temperature at which the IL phase emerges) was studied at 298 K to investigate possible hydrogen incorporation. For NPD analysis, the samples were placed in cylindrical vanadium holders with a diameter of 6 mm.

### 2.3 Diffraction Data Analysis

The analysis of SXRD and NPD data was conducted using the Rietveld refinement method implemented in the *FullProf* software suite.^33,34^ The refinement included parameters such as zero-point error, background coefficients, scale factor, asymmetry factors, lattice constants, atomic fractional coordinates (*x*,*y*,*z*), and thermal displacement factors.

### 2.4 Magnetic Properties

Magnetic properties were studied in a SQUID magnetometer (MPMS-3) from Quantum Design (San Diego, USA) through *M*(*T*) and *M*(*H*) measurements across temperatures ranging from 1.8 up to 300 K and applied external magnetic fields up to 7 T. Moreover, *ac* susceptibility was measured using the same SQUID magnetometer across frequencies ranging from 100 Hz up to 10 kHz, employing an oscillatory field with a 1 Oe amplitude.

### 2.5 X-ray Absorption Spectroscopy

Samples for X-ray absorption spectroscopy (XAS) experiments were prepared by finely grinding the powders, mixing them with cellulose, and pelletizing them into 5 mm diameter disks to optimize the absorption edge jump (∼0.52 for PrNi_0.9_Al_0.1_O_2_, reduced to 300 °C). XAS measurements were performed in the transmission mode at Ni *K*-edge (8.333 keV) using the beamline BL22-CLÆSS^35^ at ALBA Synchrotron Light Source (Cerdanyola del Vallès, Spain). A collimated X-ray beam (*H* × *V*: 1.0 × 0.5 mm^2^) was monochromatized to an energy resolution of 0.2–0.3 eV using a pair of LN_2_-cooled Si(311) crystals. The absorption coefficient was determined by measuring the photon flux with ionization chambers placed before and after the pelletized samples. Low-temperature measurements were conducted by using a helium-flow cryostat over a temperature interval of 20–290 K.

### 2.6 Absorption Data Analysis

The data reduction process was carried out using *Athena* software.^36^ This process determines the edge position *E*_0_ from the first derivative of the XANES spectrum, subtracting the pre-edge background, and performing background subtraction in the postedge range. These steps enabled normalization of the edge-jump and extraction of the EXAFS oscillations χ­(*k*) from the absorption coefficient μ­(*k*). EXAFS data were fitted using *Artemis* software.^36^ The theoretical scattering paths of the tetragonal phase (space-group: *P*4/*mmm*) of the infinite-layer compound were calculated using *FEFF* software.^37,38^ For the fitting procedure, the Fourier transforms (FT) of EXAFS functions were performed using a Hanning-type window. The window size was set in both *k*- and *R*-spaces to Δ*k* = 2.2–12.6 Å^–1^ and Δ*R* = 1.2–3.4 Å, respectively. The fitting parameters comprise the average pair distances and their bond variances, and the coordination numbers were kept fixed. The amplitude reduction factor (*S*_0_^2^ ≈ 0.704) was obtained from the fit of the Ni foil EXAFS spectrum.

### 2.7 ^1^H MAS NMR Spectroscopy of PrNi_0.9_Al_0.1_O_2_

Single pulse ^1^H magic-angle spinning (MAS) NMR experiments were conducted on a Bruker AVANCE-400 NMR spectrometer with a 9.4 T wide-bore superconducting magnet. ^1^H resonance frequency is 400.13 MHz. The powder sample was spun in a 4 mm probe at 10 kHz around an axis inclined 54°44′ with respect to the external magnetic field. The spectrum was obtained with a π/2 (5 μs) pulse and relaxation delays of 5 and 400 scans. Chemical shifts were referred to as those of the tetramethylsilane (TMS) reference.

## 3 Results

### 3.1 Initial Characterization

The samples of oxidized PrNiO_3_ and PrNi_0.9_Al_0.1_O_3_ perovskites and their reduced counterparts were prepared as black polycrystalline powders with well-formed crystalline structures. In Figure 1, the changes observed in the XRD patterns (using Cu Kα radiation) are illustrated for the PrNi_0.9_Al_0.1_O_3_ perovskite during the progressive reduction process at increasing temperatures.

**Figure 1:**
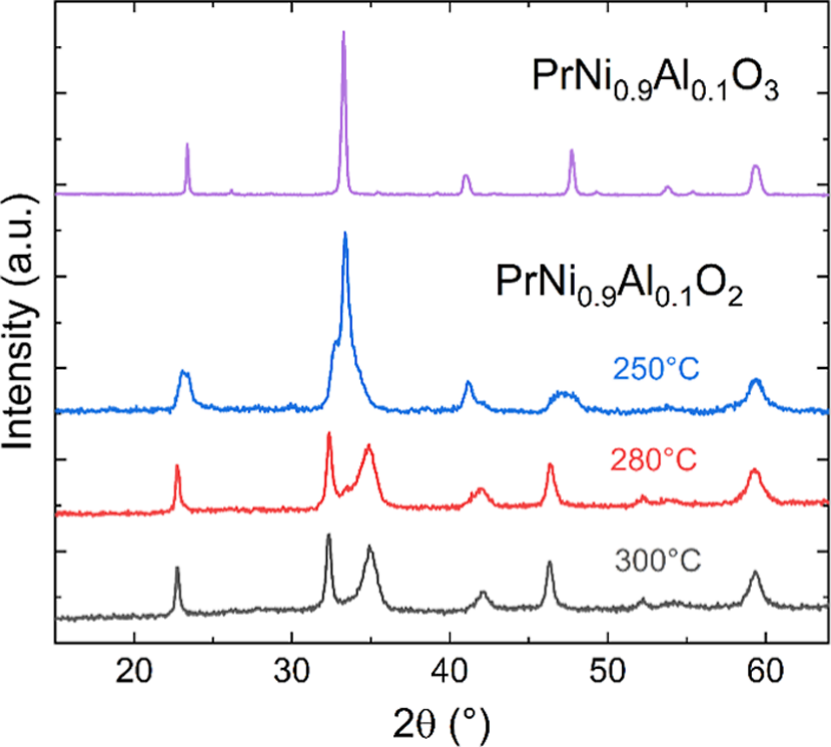
Evolution of the room-temperature XRD patterns of PrNi_0.9_Al_0.1_O_3_ (with Cu Kα radiation) upon chemical reduction with CaH_2_ at different reduction temperatures: 250 °C (blue), 280 °C (red), and 300 °C (gray).

The transformation to the infinite-layer phase appears incomplete below ∼280 °C, which is required for the reaction between the initial oxidized perovskite and CaH_2_. Preliminary Rietveld refinements against the XRD patterns for the oxidized and reduced phases are presented in Figure S1 of the Supporting Information. The oxidized perovskite phases (O3) displayed orthorhombic symmetry (space group: *Pbnm*), consistent with previous findings.^21,22^ Refined unit-cell parameters were determined as *a* = 5.419(1) Å, *b* = 5.377(1) Å, and *c* = 7.629(2) Å for PrNiO_3_, while for PrNi_0.9_Al_0.1_O_3_, they were *a* = 5.416(2) Å, *b* = 5.374(2) Å, and *c* = 7.632(3) Å. Corresponding unit-cell volumes were 222.3(1) and 222.1(1) Å^3^, respectively, indicating a slight reduction in unit-cell size with Al-substitution at the Ni sites. The reduced phases (O2) with compositions PrNiO_2_ and PrNi_0.9_Al_0.1_O_2_ exhibited tetragonal symmetry (space group: *P*4/*mmm*), with unit-cell parameters *a* = 3.940(1) Å, *c* = 3.292(1) Å, and *a* = 3.923(2) Å, *c* = 3.408(2) Å, respectively. The unit-cell volumes were 51.12(4) Å^3^ for PrNiO_2_ and 52.43(4) Å^3^ for the Al-substituted phase. Interestingly, Figure S1c,d, illustrating the Rietveld plots of the PrNiO_2_ and PrNi_0.9_Al_0.1_O_2_ reduced phases, clearly exhibits that the former is partially decomposed since the XRD pattern contains significant amounts of Pr_2_O_3_ and Ni metal, whereas the Al-doped sample only contains the IL phase, thus assessing that the presence of Al is indeed invaluable for the stabilization of the mentioned infinite-layer compound. Hereafter, the structural characterization of the reduced specimen from SXRD or NPD data is restricted to only the Al-containing specimen.

### 3.2 Oxidized Phase

The oxidized PrNi_0.9_Al_0.1_O_3_ perovskite phase was correctly refined from SXRD data in the orthorhombic *Pbnm* space group, in agreement with the structure defined for the undoped phase PrNiO_3_.^39^ Moreover, this phase is isostructural to NdNi_0.9_Al_0.1_O_3_ ^40^ but differs from the LaNi_0.9_Al_0.1_O_3_ specimen,^12^ which crystallizes in the rhombohedral *R*3̅*c* space-group.^41^ In the *Pbnm* space group, the praseodymium atoms are in the 4*c* (*x*, *y*, 0.25) Wyckoff site, while nickel and aluminum atoms are statistically distributed at the 4*b* (0.5, 0, 0) site. The oxygen anions occupy the 4*c* (*x*, *y*, 0.25) and 8*d* (*x*, *y*, *z*) positions. Rietveld refinement from SXRD data for the oxidized phase is plotted in Figure S2, and the main crystallographic results are listed in Table S1. The synchrotron high-resolution data allow for the determination of more precise unit-cell parameters: *a* = 5.41758(4) Å, *b* = 5.37407(4) Å, *c* = 7.62315(6) Å, and *V* = 221.944(3) Å^3^. Additionally, SXRD patterns for PrNi_0.9_Al_0.1_O_3_ were collected at higher temperatures up to 1173 K, revealing a phase transition from *Pbnm* to *R*3̅*c* above 773 K, as described in the Supporting Information (Figures S3 and S4 and Table S2).

On the other hand, the NPD pattern was also successfully refined using this orthorhombic model, as illustrated in Figure 2. The main crystallographic data for the oxidized phase are listed in Table 1. A minor amount of NiO was detected and included in the refinements. After achieving a satisfactory fit, the Ni/Al occupation ratio was checked; the results, within experimental errors, were consistent with the expected values. The current Al-doped perovskite exhibits a slight reduction in all three unit-cell parameters compared to the pristine PrNiO_3_ reported by Lacorre et al.^39^ from NPD data. This reduction is attributed to the smaller ionic radius of Al^3+^ (*r*_Al^3+^_ = 0.535 Å) compared to that of Ni^3+^ (*r*_Ni^3+^_ = 0.56 Å, in a low-spin configuration).

**Figure 2:**
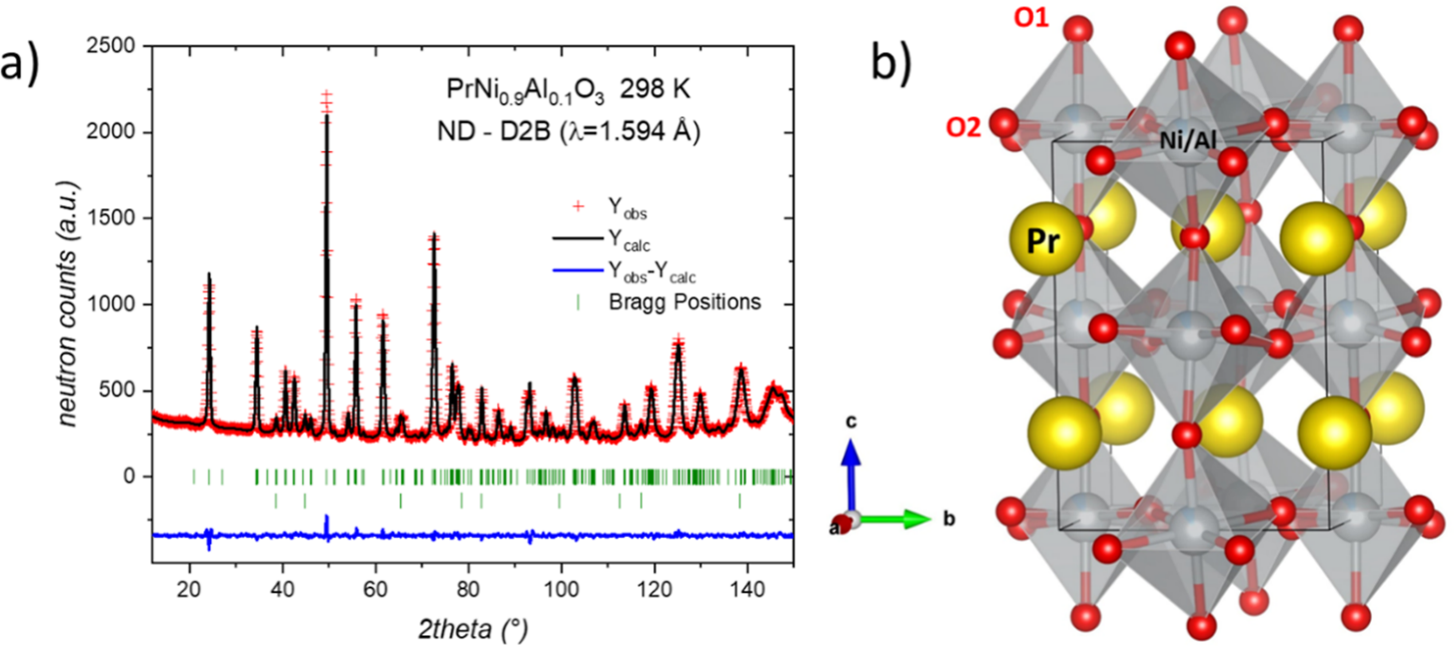
(a) Rietveld refinement of the NPD pattern at room temperature for PrNi_0.9_Al_0.1_O_3_ (a). Observed (red crosses) and calculated (black line) NPD profiles. Two series of Bragg reflections (green ticks) denote the orthorhombic phase (*Pbnm*) and NiO. (b) Schematic view of the orthorhombic crystal structure of PrNi_0.9_Al_0.1_O_3_.

**Table 1:** Main Crystallographic Results of PrNi_0.9_Al_0.1_O_3_ from NPD Data at Room Temperature^a^

orthorhombic symmetry, space-group *Pbnm* with unit-cell parameters: *a* = 5.4137(2) Å, *b* = 5.3698(2) Å, *c* = 7.6163(2) Å, and *V* = 221.41(1) Å^3^.					
atom	*x*	*y*	*z*	*U*_iso_ (Å^2^)	Occ. (<1)
Pr	1.0002(9)	0.0272(6)	0.25	0.0061(5)	
Ni	0.5	0	0	0.0021(4)	0.9
Al	0.5	0	0	0.0021(4)	0.1
O1	0.0671(5)	0.4920(7)	0.25	0.0073(7)	
O2	0.7226(3)	0.2790(3)	0.0339(2)	0.0044(6)	

a Impurity: NiO (1.4% w/w). Reliability factors: *R*_p_ = 1.89%, *R*_wp_ = 2.40%, *R*_exp_ = 1.93%, *R*_Bragg_ = 1.67%, χ^2^ = 1.54.

To study the onset of the reduction process, the intermediate product from the reactions of the perovskite PrNi_0.9_Al_0.1_O_3_ and CaH_2_, treated at 180 °C, was analyzed from NPD. The resulting diffraction pattern showed no significant changes compared to the pristine phase and was successfully refined within the orthorhombic *Pbnm* space group, as represented in Figure S5. The perovskite treated at 180 °C exhibits subtle changes, including expansion along all three crystallographic axes and the formation of oxygen vacancies at the O1 site. Although these variations are close to experimental error, these changes are aligned with the initial stage of the reduction process, as shown in Table S3. Besides, in [(Ni_0.9_Al_0.1_)­O_6_] octahedra, while the average bond lengths remain unchanged, an increase in the octahedral distortion was observed. Considering this fact, difference Fourier maps (DFM) were calculated to locate plausible hydride ions in the perovskite structure. The DFM exhibited a subtle nuclear density mismatch; however, they were not consistent with the presence of crystallographically ordered H atoms.

### 3.3 Reduced Infinite-Layer Phase

SXRD data at room temperature of the IL phase PrNi_0.9_Al_0.1_O_2+δ_ confirm the tetragonal symmetry in the *P*4/*mmm* space group, agreeing with lanthanum and neodymium analogs,^12,40^ as well as with the undoped PrNiO_2_ product synthetized via a similar route.^42^ A minor amount of Ni was detected and added during the refinements. The Rietveld refinement from the high-resolution SXRD data leads to the unit-cell parameters: *a* = 3.93009(6) Å, *c* = 3.40846(8) Å, and *V* = 52.646(2) Å^3^. This unit-cell parameter *a* is similar to that reported for another bulk PrNiO_2_ (*a* = 3.9403(3) Å)^42^ and also for the compound growth as a thin-film (*a* ≈ 3.92 Å).^5^ However, the cell parameter *c* shows a larger deviation since the reported results range from *c* = 3.2845(8) Å for the bulk specimen^42^ to *c* ≈ 3.31 Å for the thin-film,^5^ revealing an expansive effect of the unit cell probably related to the presence of Al in the structure. As observed in the lanthanum counterpart, LaNi_0.9_Al_0.1_O_2_, the diffraction pattern of this phase displays anisotropic peak broadening, which was modeled following the same approach used for this phase.^12^ The strain is more significant along the *c*-axis than within the *a*–*b* plane, likely due to stacking faults characteristic of this layered structure. Figure S6 presents the Rietveld refinement from the SXRD data for the IL phase, while Table S4 provides the corresponding crystallographic parameters. Despite the advantages of SXRD data in terms of resolution, they do not allow for the precise determination of the interlayer oxygen occupancy, highlighting the indispensable role of neutron diffraction.

The NPD pattern was properly refined, confirming the tetragonal structure. In the *P*4/*mmm* space group, the nickel/aluminum atoms are at the 1*a* (0, 0, 0) Wyckoff site, while the Pr atoms are in the 1*d* (0.5, 0.5, 0.5) site. The oxygen atoms occupy the 2*f* (0.5, 0, and 0) site. Notably, the 1*b* site, which would normally complete the perovskite-type structure, contains only about 10% oxygen atoms. In Figure 3a, the excellent agreement achieved for the NPD pattern at room temperature is shown, while in Figure 3b, a schematic view of the tetragonal crystal structure for the PrNi_0.9_Al_0.1_O_2+δ_H_γ_ phase is exhibited. Table 2 lists the main crystallographic data for this phase. The inclusion of H atoms will be detailed next. This phase was found to be stable for temperatures down to 3 K. The Rietveld fits at 150 and 3 K are included in Figure S7, and crystallographic results are listed in Tables S5 and S6.

**Figure 3:**
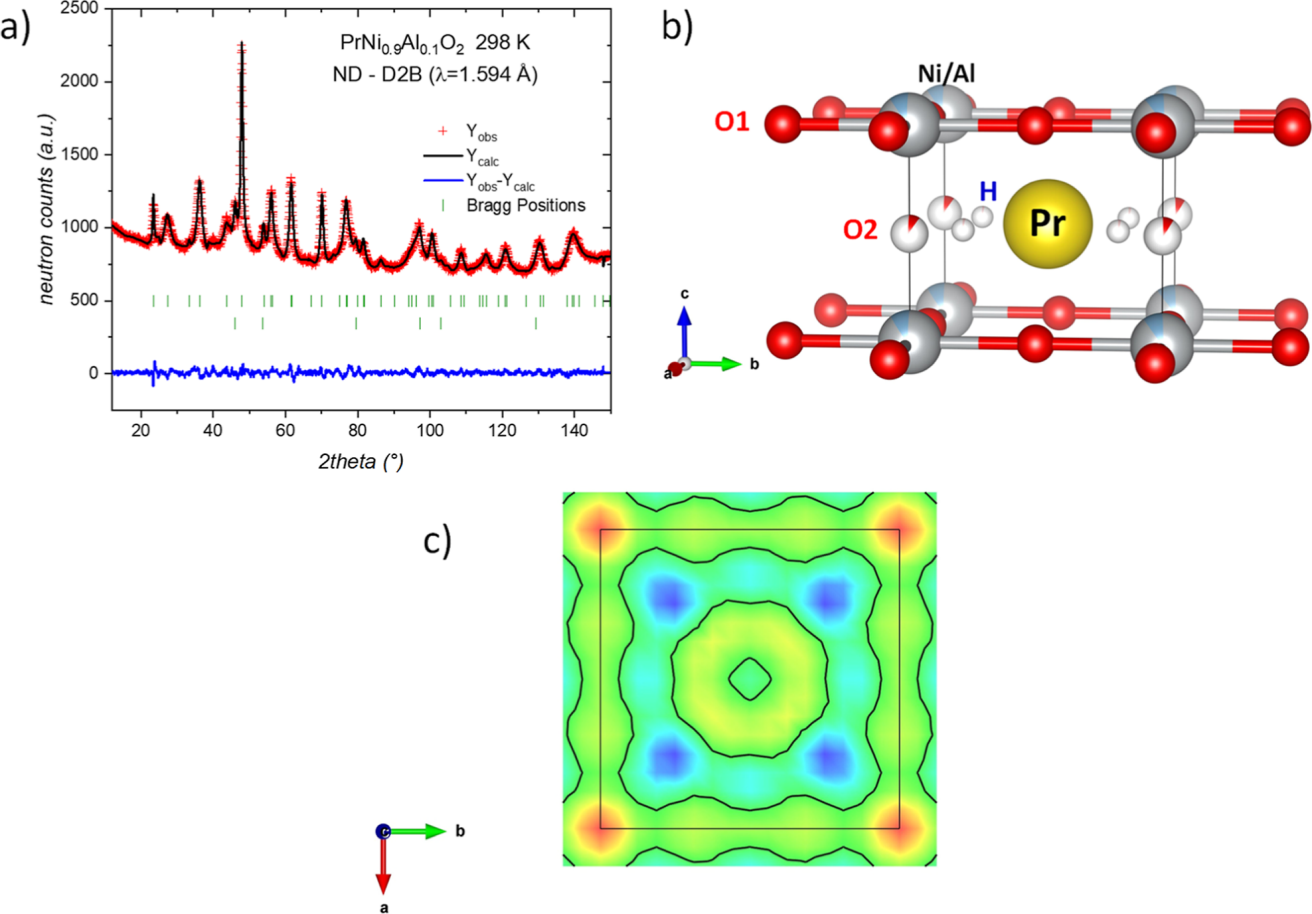
(a) Rietveld refinement from NPD data at room temperature for PrNi_0.9_Al_0.1_O_2_. Observed (red crosses) and calculated (black line) NPD profiles. Two series of Bragg reflections (green ticks) denote the tetragonal phase (*P*4/*mmm*) and Ni. (b) Schematic representation of the crystal structure of the infinite-layer phase. (c) Two-dimensional plot of the difference Fourier map in the (001) plane at *c* = 0.5.

**Table 2:** Main Crystallographic Results of PrNi_0.9_Al_0.1_O_2_ from NPD Data at Room Temperature^a^

tetragonal symmetry, space-group *P*4/*mmm*, with unit-cell parameters: *a* = 3.9270(2) Å, *c* = 3.3795(4) Å, and *V* = 52.12(1) Å^3^.						
atom	site	*x*	*y*	*z*	*U*_iso_ (Å^2^)	Occ. (<1)
Pr	1*d*	0.50000	0.50000	0.50000	0.00127	
Ni	1*a*	0.00000	0.00000	0.00000	0.00127	0.90000
Al	1*a*	0.00000	0.00000	0.00000	0.00127	0.10000
O1	2*f*	0.50000	0.00000	0.00000	0.0064(5)	
O2	1*b*	0.00000	0.00000	0.50000	0.01267	0.095(9)
H	4*k*	0.188(8)	0.188(8)	0.50000	0.01267	0.039(5)

a Impurity: Ni (3.15% w/w). Reliability factor: *R*_p_ = 1.37%, *R*_wp_ = 1.78%, *R*_exp_ = 1.36%, *R*_Bragg_ = 0.74%, χ^2^ = 1.71.

As observed for the SXRD diagrams, the NPD pattern for the IL phase also exhibits significant anisotropic peak broadening effects, which were modeled using the method previously reported by our group elsewhere.^12,40^ Similarly, initial refinements suggest that strain effects are considerably more pronounced along the *c*-axis compared to the *ab* plane, likely due to stacking faults in this layered phase. The occupancy factor of the interlayer oxygen atoms at the 1*b* (0, 0, 0.5) Wyckoff site was also refined, as shown in Table 2. Besides, small amounts of metallic nickel were detected.

A potential characteristic of infinite-layer nickelates is the incorporation of hydrides within their crystal structure. In a recent study, we demonstrated the presence of crystallographically ordered hydrogen within the unit-cell of the IL LaNi_0.9_Al_0.1_O_2_ structure.^12^ Using this finding as a reference, difference Fourier maps (DFM) were calculated and analyzed for the present infinite-layer sample at room temperature and 3 K. A detailed inspection revealed pronounced negative densities (in terms of neutron scattering length) at the (*x*, *x*, 0.5) position, with *x* ≈ 0.19, corresponding to 4*k* Wyckoff site. In Figure 3c, the negative isosurface and the color contour density in the (0, 0, and 0.5) plane are displayed, highlighting the potential hydrogen location as blue regions. Therefore, the DFM results indicate the possible presence of crystallographically ordered hydrides within the IL structure. After that, the Rietveld refinement was remade, including a hydrogen atom at the 4*k* Wyckoff site and refining the occupancy factors for both H and O2 positions. The occupancy factors for the interlayer oxygen atoms (O2) at the 1*b* (0, 0, and 0.5) sites and interstitial hydrides at 4*k* (*x*, *x*, and 0.5) yield an occupancy level of 9.5(9)% and 3.9(5)%, respectively. Consequently, the crystallographic formula of the infinite-layer phase at room temperature was found to be PrNi_0.9_Al_0.1_O_2.10_H_0.16_. Therefore, this result indicates an oxidation state of +1.18 for Ni, suggesting a high degree of nickel reduction in this sample, which approaches the expected monovalent nickel state. As shown in Figure 3b, the hydride ions are within the layers, located at 1.73(4) Å away from Pr atoms; similar La–H distances (1.7290(2) Å) were observed in LaNi_0.9_Al_0.1_O_2.11_H_0.07_ IL compound.^12^ The statistical distribution of both H and O2 ions implies an improbable coincidence of both anions in the same unit cell. It is worth highlighting the different location of H^–^ ions in the Pr vs La IL phases; the smaller *c*-axis of the Pr compound destabilizes the preferred location observed for La, in the center of Ni–O squares.^12^

As complementary evidence of the presence of H in the sample, we have collected a ^1^H nuclear magnetic resonance (NMR) spectrum of the LaNi_0.9_Al_0.1_O_2.11_H_0.07_ IL compound. The single pulse ^1^H magic-angle spinning (MAS) NMR spectrum is included in the Supporting Information as Figure S8. The observed signal from ^1^H is strong, and it is convoluted with the paramagnetic field of Pr, indicating that H and Pr are tightly bonded. Indeed, our NPD data show that H atoms are coordinated to Pr in [PrH_4_] units, as shown in Figure 3b, with H located 1.73(4) Å from Pr atoms.

### 3.4 X-ray Absorption Spectroscopy

The valence states of Ni cations in infinite-layer PrNi_0.9_Al_0.1_O_2+δ_ (as reduced at 300 °C during 30 min) were probed utilizing XANES data at the Ni *K*-edge, recorded under room conditions. In Figure 4a, the normalized XANES spectrum of the IL nickelate is plotted together with the spectra of PrNi_0.9_Al_0.1_O_3_ and metallic nickel. In fact, the reduced sample presented an edge shift to lower energy by ∼2.1 eV (as indicated by vertical bars in Figure 4a) from the edge position of oxidized nickelate (O3). A similar energy shift of ∼1.7 eV was observed in NdNi_0.9_Al_0.1_O_2_ counterpart,^40^ confirming the transition in the nickel valence from +3 to +1. A gradual decrease in white line intensity just above the absorption edge energy could be detected as the IL phase is formed, which potentially indicates a reduction of the density of available unoccupied electronic states for the Ni core electrons and denotes a more metallic tendency for PrNi_0.9_Al_0.1_O_2_.

**Figure 4:**
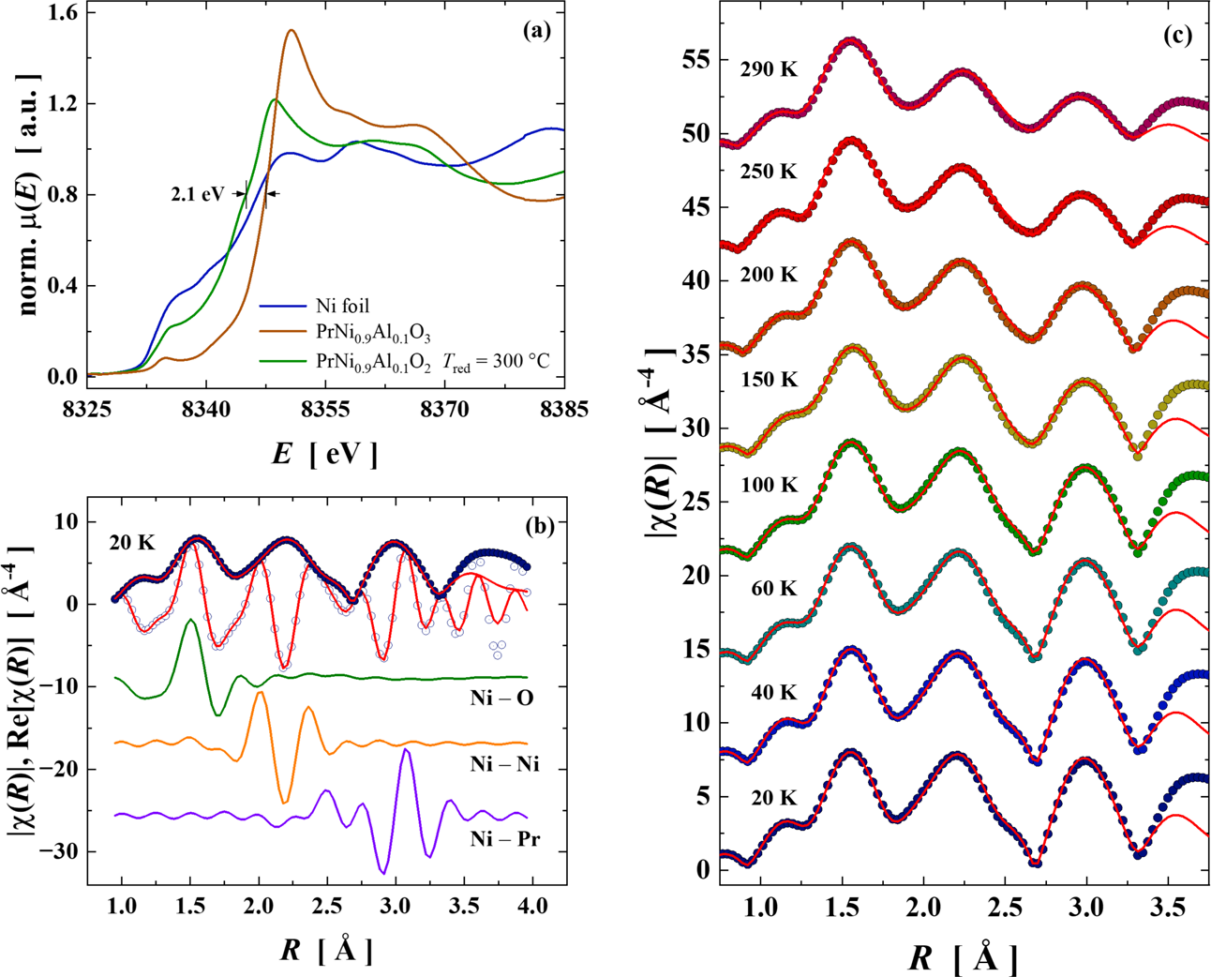
(a) Raw normalized Ni *K*-edge XANES spectra of metallic Ni, oxidized PrNi_0.9_Al_0.1_O_3_, and reduced PrNi_0.9_Al_0.1_O_2_ recorded under room conditions. (b) The edge energy is defined as the point on the rising of the absorption spectrum where the intensity reaches 0.8 of the edge jump. Fourier transform of *k* ^3^χ­(*k*) raw data recorded at 20 K (open symbols), along with the individual scattering paths (colored lines) and their summed contributions (red lines). (c) Temperature-dependent EXAFS data showing the Fourier transform magnitude of *k* ^3^χ­(*k*) in *R* space are shown for PrNi_0.9_Al_0.1_O_2_.

In depth knowledge of atomic rearrangements around Ni atoms was assessed by Ni *K*-edge EXAFS measurements. First, a local analysis was conducted in IL PrNi_0.9_Al_0.1_O_2_ at the lowest temperature achieved (20 K) to ensure a reliable structural model as low temperature minimizes artifacts caused by low signal-to-noise effects at room temperature. In Figure 4b, the raw Fourier transform of the *k* ^3^-weighted *k* ^3^χ­(*k*) extracted EXAFS oscillation is represented as magnitude |χ­(*R*)| and real part *Re*[χ­(*R*)] (open symbols). The EXAFS signal was properly described by a local structural model considering three single scattering paths: Ni–O, Ni···Ni, and Ni···Pr. In the IL tetragonal structure, the first coordination environment, Ni–O, forms a square planar unit [NiO_4_] (*N*_*j*_ = 4). The next path allowed in this system concerns Ni···Pr with a coordination number *N*_*j*_ of 8, which forms the cation sublattice. However, an additional path occurred in the radial range 2.0–2.5 Å. Like the observation in IL NdNi_0.9_Al_0.1_O_2_,^40^ such a component was assigned to the most intense Ni···Ni path (*N*_*j*_ = 12), originating from metallic nickel stabilized after the reduction process at *T*_red_ = 300 °C. Table 3 summarizes the local structure parameters (average path distance and bond variance) extracted from the EXAFS data recorded at 20 K.

**Table 3:** Local Structure Parameters from Ni *K*-Edge EXAFS Fitting at 20 K for PrNi_0.9_Al_0.1_O_2_ ^a^

	EXAFS of PrNi_0.9_Al_0.1_O_2_ at 20 K			parameters from Einstein fitting		
SS path	*d*_*j*_ (Å)	σ_*j*_^2^ (10^–3^ Å^2^)	*N*_*j*_	θ_E_ (K)	σ_s_^2^ (10^–3^ Å^2^)	κ_E_ (eV Å^–2^)
Ni–O	1.965(7)	4.8(3)	4	765	2.4	13.1
Ni···Ni	2.480(5)	6.7(1)	12	330	4.4	5.7
Ni···Pr	3.249(7)	8.1(3)	8	220	5.7	3.6
	*R*-factor	0.0049				
	Δ*k* (Å^–1^)	10.4	*N*_*idp*_	14		
	Δ*R* (Å^–1^)	2.2	*N*_v_	10		

a Abbreviations: *d*_*j*_ is the average path distance, σ_*j*_^2^ is the bond variance, *N*_*j*_ is the coordination number, θ_E_ is the Einstein temperature, σ_s_^2^ is the static disorder, κ_E_ is the effective force constant, *N*_idp_ is the number of independent variable from the relation 2Δ*k*·Δ*R*/π, and *N*_v_ is the free variable used for EXAFS fitting.

Then, the local atomic structure at Ni sites was evaluated considering EXAFS data under temperature variations ranging from 20 K up to 290 K. This analysis allows for precise probing of potential structural transitions and lattice dynamics in IL PrNi_0.9_Al_0.1_O_2_. The temperature-dependent EXAFS spectra were fitted by using the structural model in Table 3. The fitting convergence remained stable, with the *R*-factor ranging from 0.0027 to 0.0077 for PrNi_0.9_Al_0.1_O_2+δ_ (see Table S7). The temperature evolution of the Fourier transform magnitude is plotted in Figure 4c. As noticed for NdNi_0.9_Al_0.1_O_2_,^40^ no abrupt changes in the shape of |χ­(*R*)| functions were observed, suggesting the absence of temperature-induced structural phase transitions.

### 3.5 Magnetic Properties

The temperature-dependent magnetic susceptibility χ­(*T*) values for PrNi_0.9_Al_0.1_O_3_ and PrNi_0.9_Al_0.1_O_2_ samples measured in both zero-field cooling (ZFC) and field-cooling (FC) conditions at the applied magnetic field of *H*_dc_ = 100 Oe are represented in Figure 5a. For the PrNi_0.9_Al_0.1_O_3_ perovskite, an antiferromagnetic (AFM)-like behavior is observed, with a minimal magnetic susceptibility. However, the reduced PrNi_0.9_Al_0.1_O_2_ IL phase reveals a significant irreversibility between ZFC/FC curves persisting until highest measured temperature (∼300 K), which can be attributed to the presence of ferromagnetic-like Ni particle impurities that segregate during the reduction process to give the infinite-layer phase.^43^ From the modified Curie–Weiss (C–W) law χ­(*T*) = χ_0_ + *C*/(*T* – Θ) (χ_0_ is a temperature-independent constant, Θ is the Weiss temperature, and C is the Curie constant),^44^ we fitted the inverse of magnetic susceptibility χ^–1^(*T*) for PrNi_0.9_Al_0.1_O_3_, displayed in Figure 5b. The fitting (red line) yields Θ = −81.2 K, *C* = 2.407(8) emu K/mol, and χ_0_ = −5.607(5) × 10^–2^ emu/mol. The negative Weiss temperature confirms the AFM interactions, as further demonstrated by the isotherms *M*(*H*) curves (Figure 5c) at different temperatures (1.8, 30, and 300 K), exhibiting typical linear behavior. The *C* value gives a paramagnetic moment of 4.39 μ_B_/f.u (from $\mu =\sqrt{8C}$ ). This indicates that the magnetic interactions in PrNi_0.9_Al_0.1_O_3_ are mainly between Pr^3+^ (*J* = 4) and Ni^3+^ (*S* = 3/2) in the high spin state, with a theoretical magnetic moment of 5.13 μ_B_.

**Figure 5:**
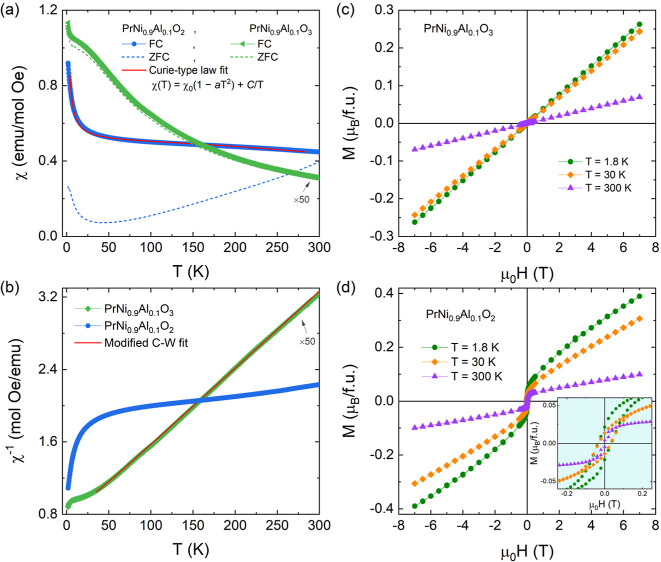
(a) Temperature dependence of the magnetic susceptibility χ­(*T*) at FC (symbol) and ZFC (dashed line) protocols for the PrNi_0.9_Al_0.1_O_3_ and PrNi_0.9_Al_0.1_O_2_ samples under the external magnetic field of *H*_dc_ = 100 Oe. The red line represents the fit by the Curie-type law χ­(*T*) = χ_0_(1 – *aT* ^2^) + *C*/*T*. (b) χ^–1^(*T*) curves for both samples. The red line represents the fitting with modified C–W law for PrNi_0.9_Al_0.1_O_3_. (c,d) Isotherms *M*(*H*) curves recorded at 1.8, 30, and 300 K for both samples.

Differently, the reduced sample PrNi_0.9_Al_0.1_O_2_ does not show a typical linear response of the χ^–1^(*T*) curve (see Figure 5b). As observed in Figure 5d, the *M*(*H*) curves reveal a ferromagnetic-like or superparamagnetic sigmoid behavior at lower fields, which can be associated with the presence of elemental Ni particles segregated in the reduction process,^11^ similar to that previously reported for LaNi_0.9_Al_0.1_O_2_.^12^ In this case, we fitted the χ­(*T*) curve by the Curie-type law χ­(*T*) = χ_0_(1 – *aT* ^2^) + *C*/*T* (see red line in Figure 5a), including Pauli and van Vleck paramagnetism.^43^ The fit yields *a* = 2.094(4) × 10^–8^ 1/K^2^, *C* = 1.411(1) emu K/mol, and χ_0_ = 0.497(3) emu/mol, which is like those observed for La_1–*x*_Ca_*x*_NiO_2+δ_ infinite-layer polycrystal.^43^ The *C* value gives a paramagnetic moment of 3.36 μ_B_/f.u, being lower than that for the oxidized PrNi_0.9_Al_0.1_O_3_ perovskite, due to the reduction of Ni^3+^ to Ni^+^, which has a lower spin value of *S* = 1/2.

## 4 Discussion

### 4.1 Anisotropic Thermal Expansion and Electron Count

As mentioned in Section 3.3, the tetragonal symmetry remains stable down to 3 K, from the NPD data. In Figure S9, the unit-cell parameters and volume as a function of temperature are plotted for PrNi_0.9_Al_0.1_O_2_. In fact, all of the parameters exhibit a typical expansion with increasing temperature; however, it is notable that the linear expansion along the *c*-axis is higher than that observed along the *a*- and *b*-axes. The thermal expansion coefficients (TEC) are 6.2 × 10^–6^/K and 20.5 × 10^–6^/K along *a*-/*b*-axis and *c*-axis, respectively (Table 4). This behavior can be understood considering the layered nature of the tetragonal phase, where the intralayer interactions are much stronger than those between adjacent layers. To delve deeper into this aspect, the evolution of the unit-cell parameters of LaNi_0.9_Al_0.1_O_2_ and NdNi_0.9_Al_0.1_O_2_ infinite-layer phases is compared to PrNi_0.9_Al_0.1_O_2_ in Figure 6a. The TEC for these phases in the 3–298 K temperature range along *a*- and *c*-directions are also compared in Figure 6b. Regarding the unit-cell parameters, it is evident that *a* gradually decreases as the ionic radii of the lanthanides decrease, following the order: La^3+^ > Pr^3+^ > Nd^3+^. In contrast, the *c* parameter for the present phase exhibits a significantly smaller value than those observed for the La and Nd phases. A similar comparison can be established with the TEC values, where the Pr phase exhibits a higher TEC along the *c*-axis. This distinct behavior can be attributed to the nearly complete elimination of interlayer oxygen atoms in the praseodymium phase. Another peculiar characteristic of the Pr phase is the average oxidation state of nickel, which is the highest in the aluminum-doped IL family *R*Ni_0.9_Al_0.1_O_2+δ_. Based on the crystallographic stoichiometry obtained from NPD, as listed in Table 4, the average oxidation state of nickel in *R*Ni_0.9_Al_0.1_O_2+δ_ is +1.10, +1.18, and +1.16 for *R* = La, Pr, and Nd, respectively. We can recall here that the oxidation state of nickel that replicates the electronic configuration of superconducting cuprates is nominally Ni^1+^, which corresponds to a *d* ^9^ electronic configuration. More precisely, for the PrNiO_3_ thin films, a superconducting dome has been reported,^6^ which appears when the oxidation state of nickel is between Ni^1.12+^ and Ni^1.23+^. This, when we use terms of electronic count, means that superconductivity should appear between *d* ^8.88^ and *d* ^8.77^ electronic configurations. However, our polycrystalline specimen with an experimentally determined nickel valence of +1.16 shows no signs of superconductivity. The incorporation of hydrogen could thus be related to the absence of superconductivity in this compound.

**Table 4:** TEC for La, Pr, and Nd Infinite-Layer Phases (RNi_0.9_Al_0.1_O_2+δ_) Calculated from the Temperature Evolution of Unit-Cell Parameters

	TEC (×10^–6^/K)				
RNi_0.9_Al_0.1_O_2_	a-axis	c-axis	overall	crystallographic formula	refs
*R* = La	5.3	14.0	8.2	LaNi_0.9_Al_0.1_O_2.11_H_0.07_	^12^
*R* = Pr	6.2	20.5	11.0	PrNi_0.9_Al_0.1_O_2.10_H_0.16_	present work
*R* = Nd	5.0	10.2	6.7	NdNi_0.9_Al_0.1_O_2.17_	^40^

**Figure 6:**
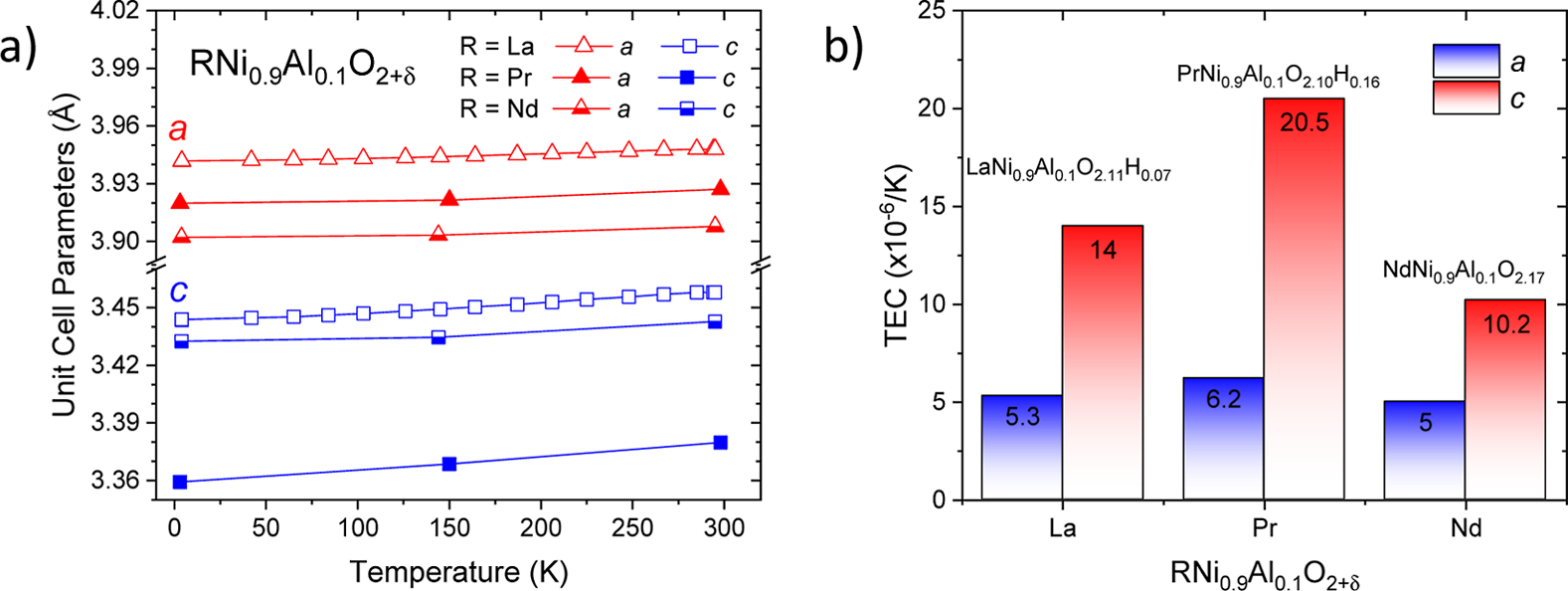
Unit-cell parameters and cell volume versus temperature (a) and TEC for *a* and *c* parameters (b) for *R* = La,^12^ *R* = Pr, and *R* = Nd^40^ infinite-layer nickelate phases (*R*Ni_0.9_Al_0.1_O_2_).

### 4.2 Local Atomic Structure at Ni Sites

One of the main advantages of the EXAFS technique lies in its ability to direct probe the parallel mean square relative displacement (σ_*j*_^2^, the bond variance in units of Å^2^) between the absorber and backscatter atoms.^45–47^ This displacement takes place along the direction of the single scattering path, and it is composed of two components, as follows

1 $$\sigma_{j}^{2}=\sigma_{s}^{2}+\sigma_{d}^{2}$$

where σ_s_^2^ stands for the static disorder, and it is a temperature-independent parameter, while the σ_d_^2^ depends on the thermal motions induced under temperature variations, and it can be linked to the density of vibrational states. Within the harmonic approximation, where there is no appreciable change along the average path distance (Table S7 lists the obtained path distances, elucidating that maximum relative deviations for paths Ni–O, Ni···Ni, and Ni···Pr under temperature variation are ∼0.23%, 0.04%, and 0.05%, respectively), the second contribution can be described by the Einstein model,^21,47–49^ as given by

2 $$\sigma_{d}^{2}=\frac{\hbar^{2}}{2\mu k_{B}\theta_{E}}\;\coth\left[\frac{\theta_{E}}{2T}\right]$$

where θ_E_ represents the Einstein temperature that is linked to the Einstein frequency [ω_E_ = (*k*_B_/ℏ)­θ_E_], μ is the reduced mass of the atomic pair (here, Ni–O, Ni···Ni, or Ni···Pr), and (*k*_B_, ℏ, *T*) maintain their usual physical meaning. In Figure 7, the bond variances for paths Ni–O, Ni···Ni, and Ni···Pr are plotted together with the respective Einstein fitted curves. In fact, we derived the Einstein temperature of 765, 330, and 220 K for Ni–O, Ni···Ni, and Ni···Pr, respectively, each of them relates to the static disorder of 2.4 × 10^–3^ Å^2^, 4.4 × 10^–3^ Å^2^, and 5.7 × 10^–3^ Å^2^ (see in Table 3).

**Figure 7:**
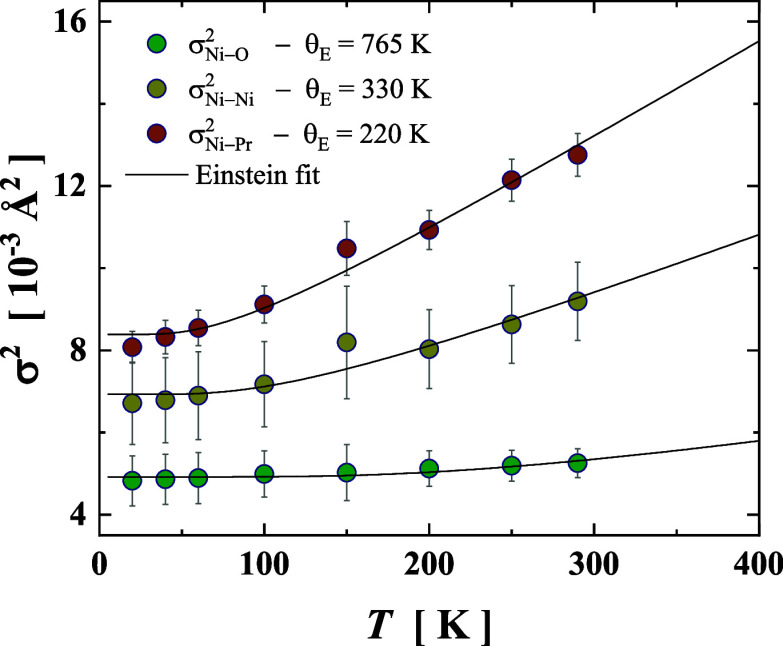
Temperature dependence of the bond variance (σ_*j*_^2^) for the single scattering paths (Ni–O, Ni···Ni, and Ni···Pr) used to describe the EXAFS oscillations of the PrNi_0.9_Al_0.1_O_2_ infinite-layer. The black lines represent the best fit to the Einstein model in eq 2.

Interestingly, the Einstein temperature for the scattering path Ni–O in PrNi_0.9_Al_0.1_O_2_ (765 K) shows a significant increase compared to PrNiO_3_ (553 K).^21^ However, the Einstein temperature for path Ni···Pr within Pr-based infinite-layer has a similar θ_E_ temperature (220 K) obtained in NdNi_0.9_Al_0.1_O_2_ (230 K).^40^ For metallic Ni, the Einstein temperature for path Ni···Ni was 330 K, a value that agrees well with those reported elsewhere for *cF*4 cubic Ni. From the Einstein temperature, the effective force constant (κ_E_) associated with the atomic pair interaction can be estimated in the harmonic approximation by the simple formulas $\kappa_{E}=\mu {\left(\frac{\theta_{E}k_{B}}{\hbar }\right)}^{2}$ ,^50,51^ listed in Table 3. For the Ni–O bond, the estimated force constant κ_E_ is approximately 13 eV Å^–2^, nearly twice the value obtained for the Ni–O bond in PrNiO_3_ (6.8 eV Å^–2^) and slightly higher than that observed in NdNi_0.9_Al_0.1_O_2_ (9.7 eV Å^–2^).^40^ On the other hand, the Ni···A sublattice (A = Pr, Nd) shows no significant change, with estimated force constants being ∼3.6 and ∼3.9 eV Å^–2^ for Pr and Nd, respectively. Therefore, PrNi_0.9_Al_0.1_O_2+δ_ exhibits a higher degree of covalency at the local level of the Ni–O bonds compared to those of both NdNi_0.9_Al_0.1_O_2+δ_ and PrNiO_3_. The Ni–O bond lies within the *a-b* plane of the *P*4/*mmm* tetragonal lattice (see Figure 3b), and this finding suggests strong lattice coupling, which could have important implications for the superconducting performance of Pr-based infinite-layer compounds.

### 4.3 Spin Glass-like Behavior

As described in Section 3.5, the difference between ZFC and FC in the susceptibility curves as well as the weak isothermal hysteresis for the reduced PrNi_0.9_Al_0.1_O_2+δ_ sample can be interpreted as the feature of a spin glass (SG)-like state, similar to those already reported for La_1–*x*_Ca_*x*_NiO_2+δ_ and *R*NiO_2_ (R = La, Pr, and Nd).^42,43^ To provide insight into the dynamics and nature of the SG phase, we have studied the *ac* susceptibility χ_*ac*_(*T*) (real part) at several fixed frequencies, as displayed in Figure 8a. As noticed before, the pronounced cusp/peak with a freezing temperature *T*_f_ ≈ 4 K shifts to higher temperatures as frequency is increased, whereas the magnitude decreases, suggesting a characteristic feature of the SG-like transition in PrNi_0.9_Al_0.1_O_2+δ_.

**Figure 8:**
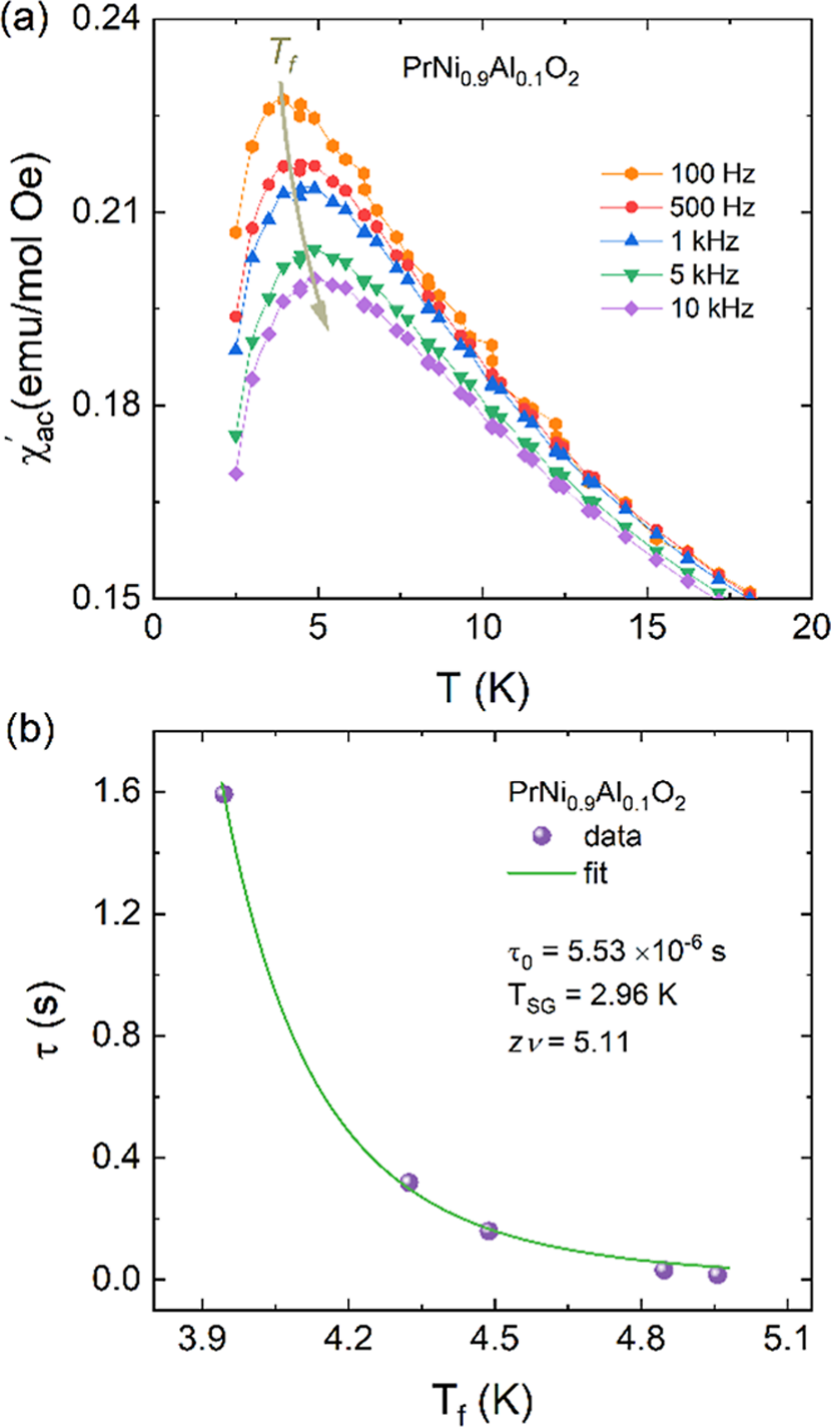
(a) Temperature dependence of the real part of *ac* susceptibility χ_*ac*_′(*T*) measured at different fixed frequencies (100 Hz up to 10 kHz) for the reduced PrNi_0.9_Al_0.1_O_2+δ_ sample. (b) Relationship between the freezing temperature *T*_f_ and time period τ = (2π*f*)^−1^ together with the best fit by eq 3.

The relaxation time τ = (2π*f*)^−1^ around the freezing temperature in a SG system is described by the following power-law

3 $$\tau =\tau_{0}{\left[\frac{T_{f}}{T_{SG}}-1\right]}^{-zv},,T_{f}>T_{SG}$$

where *T*_SG_ is the SG temperature as the *f* → 0, τ_0_ is the characteristic flipping time of single spin flip, and *zv* is the dynamical critical exponent.^52^ In Figure 8b, the relationship between the freezing temperature *T*_f_ and the time-period τ = (2π*f*)^−1^ is shown together with the best fit using the eq 3, which yielded τ_0_ = 5.56 × 10^–6^ s, *T*_SG_ = 2.96 K, and *zv* = 5.11. The critical exponent *zv* assumes the characteristic value between 4 and 13 ^52^, whereas τ_0_ is larger than those typical canonical and cluster glasses (10^–10^ to 10^–13^ s).^53^ A high value of τ_0_ indicates a slow spin dynamic in PrNi_0.9_Al_0.1_O_2+δ_. These results suggests that the spin glass state could also be described by the Vogel–Fulcher (VF) law, represented as τ = τ_0_ exp­[*E*_a_/*k*_B_(*T* – *T*_f_)] (where *E*_a_ stands for an energy barrier separating two low energy states, and *k*_B_ is the Boltzmann constant), as reported by Ortiz et al. for the infinite-layer nickelates *R*NiO_2_ (R = La, Pr, and Nd).^11^ In this case, the authors suggest that weak or intermediate coupling between magnetic clusters is responsible for the spin dynamics in the system. We have demonstrated through the local atomic structure analysis (Section 4.2) that PrNi_0.9_Al_0.1_O_2+δ_ exhibits a high local level of covalency of the Ni–O bonds, suggesting a strong lattice coupling and consequently implying a greater intercluster spin dynamics. In addition, the calculated relative shift (*k*) in the freezing temperature *k* = Δ*T*_f_/(*T*_f_Δlog_10_ *f*) yields *k* ≈ 0.12, which would be between values for canonical spin-glass and superparamagnetic systems.^42^ This further corroborates our findings shown in Section 3.5, where PrNi_0.9_Al_0.1_O_2+δ_ behaves as a superparamagnetic-like system at low fields. Therefore, we believe that the dynamics of the glassy behavior of PrNi_0.9_Al_0.1_O_2_ is mainly mediated between canonical spin glass and superparamagnetic clusters.

## 5 Concluding Remarks

We have demonstrated that Ni^+^ stabilization in defect perovskites PrNi_1–*x*_Al_*x*_O_2+δ_ is achievable under mild reduction conditions using CaH_2_, from suitable perovskite precursors of the composition PrNi_1–*x*_Al_*x*_O_3_. The reduced compounds consist of a tetragonally distorted perovskite where most of the axial oxygen atoms are eliminated, leaving Ni coordinated to four oxygens in a square-planar geometry. NPD, which is particularly sensitive to oxygen and hydrogen atoms, was used to refine the structural model based on the well-known “infinite-layer” structure typified for SrCuO_2_. In our structural refinement using NPD data, we assumed that Al cations, randomly distributed within the Ni sublattice, retain octahedral coordination, even in the reduced phase, as depicted in Figure 9.

**Figure 9:**
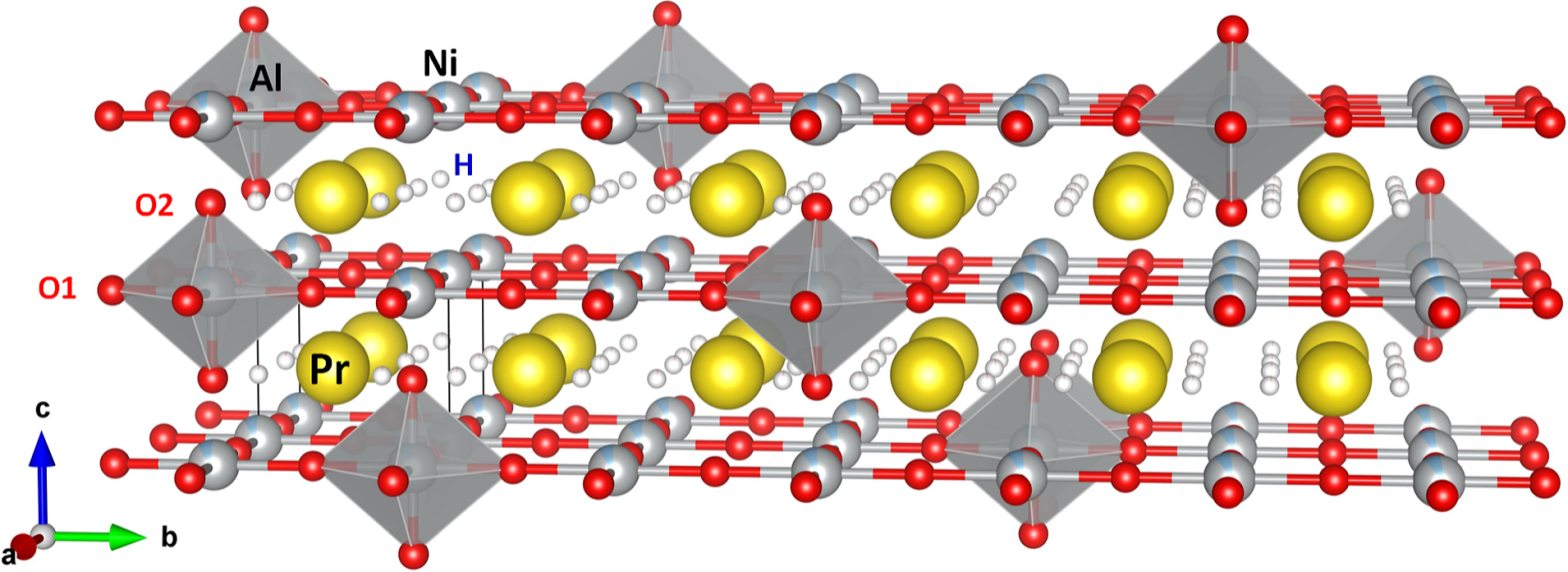
Crystal structure of PrNi_0.9_Al_0.1_O_2.1_H_0.1_, illustrating [AlO_6_] octahedra bridging adjacent layers, with Ni^+^ ions in 4-fold or 5-fold oxygen coordination, and the statistical presence of H^–^ ions positioned between two Pr atoms.

Neutron diffraction analysis also reveals the presence of excess oxygen atoms at axial positions to maintain the octahedral coordination of Al. The tetragonal structure exhibits a significantly shorter *c*-axis relative to the *a*-axis due to the absence of most axial oxygens. The incorporation of Al within the Ni sublattice therefore plays a crucial role in structural stabilization as the associated axial oxygen atoms bridge adjacent layers, there by enhancing the stability.

The presence of Ni^+^ is confirmed through XAS spectroscopic techniques, which clearly demonstrates this rare oxidation state. Magnetic measurements suggest the formation of segregated Ni metal in the reduced samples. However, synchrotron X-ray and neutron diffraction—despite the large neutron scattering length of Ni—failed to detect any segregated Ni, possibly due to its nanoparticulate form, which is below the detection limit of these diffraction techniques. Regardless, magnetic susceptibility measurements show no signs of superconductivity, either in the undoped Pr sample or in the Al-doped variant, PrNi_0.9_Al_0.1_O_2+δ_.

The presence of H^–^ ions, statistically distributed within the tetragonal unit cell at a non-negligible concentration (∼0.16 atoms per formula unit), is determined by NPD data. This topic has been the subject of considerable debate as theoretical studies suggest that hydrogen intercalation is energetically favorable and induces significant electronic structure modifications. Our NPD-based difference Fourier maps reveal a negative scattering density at midpoints between Pr atoms, consistent with hydrogen being trapped within the crystal layers. This is further confirmed by NMR results. On the other hand, the absence of superconductivity in our samples could be linked to this hydrogen inclusion, which appears to be an inherent consequence of the synthesis process in the presence of CaH_2_. However, alternative explanations should not be ruled out. Finally, local atomic structure analysis shows that PrNi_0.9_Al_0.1_O_2+δ_ exhibits a high local level of covalency of the Ni–O bonds, suggesting a strong lattice coupling.

## Supplementary Material



## Notes

Laboratory XRD, SXRD, and NPD patterns of diverse oxidized and reduced phases; structural parameters from Rietveld refinements; unit-cell parameters thermal variation; temperature-dependent EXAFS parameters; and ^1^H NMR spectra for reduced specimen (PDF)
