# Supplementary material for: From Oxidized PrNi0.9Al0.1O3 to Reduced PrNi0.9Al0.1O2+δ Perovskite Nickelates: Stabilization of Infinite-Layer Specimens with Monovalent Ni in the Bulk Polycrystalline Form
Source: Inorg Chem. 2025 Jul 22;64(30):15620–31. doi: 10.1021/acs.inorgchem.5c02051 (PMC12326351; doi:10.1021/acs.inorgchem.5c02051)
Supplement: Supplementary file 1 [file ic5c02051_si_001.pdf]

SUPPLEMENTARY INFORMATION

From oxidized $PrNi_{0.9}Al_{0.1}O_3$ to reduced $PrNi_{0.9}Al_{0.1}O_{2+\delta}$ perovskite nickelates: Stabilization of infinite-layer specimens with monovalent Ni in bulk polycrystalline form

Javier Gainza[1,2], Carlos A. López[3], Romualdo S. Silva Jr[1,4], João Elias F. S. Rodrigues[2], Federico Serrano-Sánchez[1], Alina Skorynina[5], Norbert M. Nemes[1,4], María T. Fernández-Díaz[6], José Luis Martínez[1], and José Antonio Alonso[1,†]

[(1)] *Instituto de Ciencia de Materiales de Madrid, CSIC, Cantoblanco 28049 Madrid, Spain.*

[(2)] *European Synchrotron Radiation Facility (ESRF), 71 Avenue des Martyrs, 38000 Grenoble, France.*

[(3)] *INTEQUI, (UNSL-CONICET) and Facultad de Química, Bioquímica y Farmacia, UNSL, Almirante Brown 1455, 5700, San Luis, Argentina.*

[(4)] *Departamento de Física de Materiales, Universidad Complutense de Madrid, E-28040 Madrid, Spain.*

[(5)] *CELLS−ALBA Synchrotron Light Source, Barcelona E-08290, Spain.*

[(6)] *Institut Laue Langevin, 38000 Grenoble Cedex, France.*

[†] **Corresponding Author:** José Antonio Alonso (ja.alonso@icmm.csic.es)

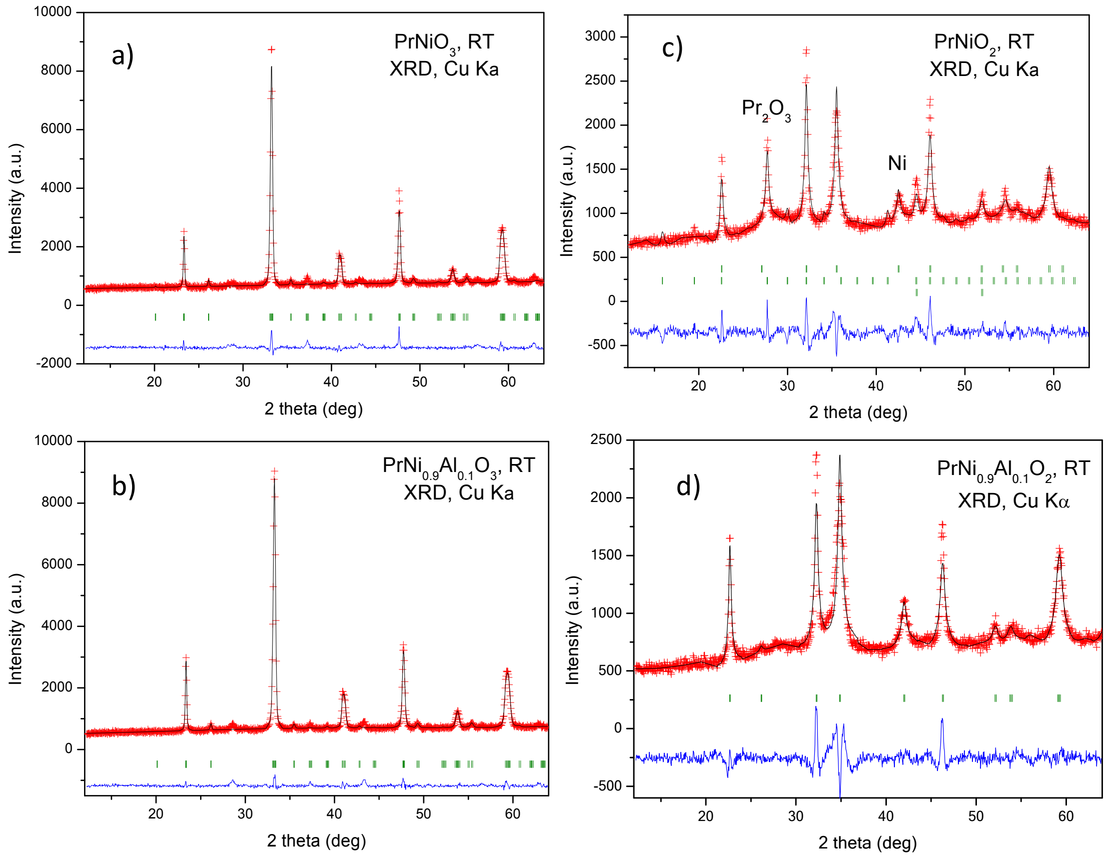

Fig. S1: Rietveld plots against laboratory XRD data with Cu-$K\alpha$ radiation. (a) Oxidized $PrNiO_3$ perovskite, defined in the $Pbnm$ space group. (b) Oxidized $PrNi_{0.9}Al_{0.1}O_3$, defined in the $Pbnm$ space group. (c) Reduced $PrNiO_2$, showing a tetragonal IL structure; the pattern contains significant amounts of Pr_2O_3 and Ni metal. (d) Reduced $PrNi_{0.9}Al_{0.1}O_2$, identified as a pure infinite-layer tetragonal phase, defined in the space group $P4/mmm$.

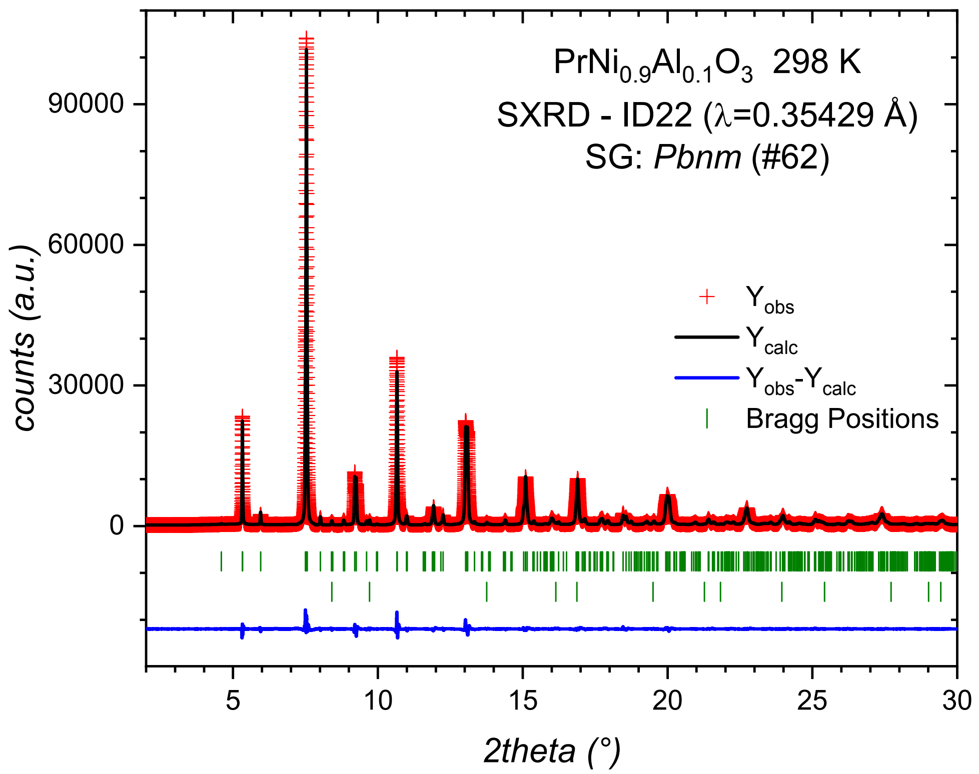

Fig. S2: (a) Rietveld refinement of the SXRD pattern at room temperature for $PrNi_{0.9}Al_{0.1}O_3$. Observed (red crosses) and calculated (black line) SXRD profile.

Table S1: Main crystallographic results of $PrNi_{0.9}Al_{0.1}O_3$ from SXRD data at room temperature.

Orthorhombic symmetry, space-group *Pbnm* with unit-cell parameters: a = 5.41758(4) Å, b = 5.37407(4) Å, c = 7.62315(6) Å, and V = 221.944(3) Å³.

**Atom**	***x***	***y***	***z***	**U_{iso} (Å²)**	**Occ. (<1)**
Pr	0.99602(8)	0.02486(3)	0.25000	0.00712(5)	
Ni	0.50000	0.00000	0.00000	0.00386(5)	0.9
Al	0.50000	0.00000	0.00000	0.00386(5)	0.1
O1	0.0613(6)	0.4950(3)	0.25000	0.0085(9)	
O2	0.7212(4)	0.2780(4)	0.0360(3)	0.0091(5)	

Impurity: NiO (1.51% w/w)
Reliability Factors: R_p = 4.55%, R_{wp} = 6.13%, R_{exp} = 0.84%, R_{Bragg} = 1.96%, χ^2 = 53.1

High temperature crystal structure of $PrNi_{0.9}Al_{0.1}O_3$

The high temperature evolution of crystal structure was studied using SXRD collected at selected temperatures above RT: 473, 673, 773, 873, 973, 1073, and 1173 K. The thermal evolution of the SXRD patterns reveals a phase transition as illustrated in **Fig. S3a** for selected diffraction lines. Rietveld refinements show that a pure orthorhombic phase is observed at 473 K, however, above 773 K the rhombohedral $R\bar{3}c$ phase is already present. This model agrees with that observed for the lanthanum counterpart $LaNi_{0.9}Al_{0.1}O_3$ at room temperature [1]. **Fig. S4** plots the Rietveld refinement of the pattern collected at 1173 K and **Table S2** lists the main crystallographic results at 1173 K. At intermediate temperatures, 673 and 773 K, the patterns exhibit a mixture of both orthorhombic and rhombohedral phases. The thermal evolution of the phase composition and the unit-cell parameters are plotted in **Fig. S3b** and **Fig S3c**, respectively.

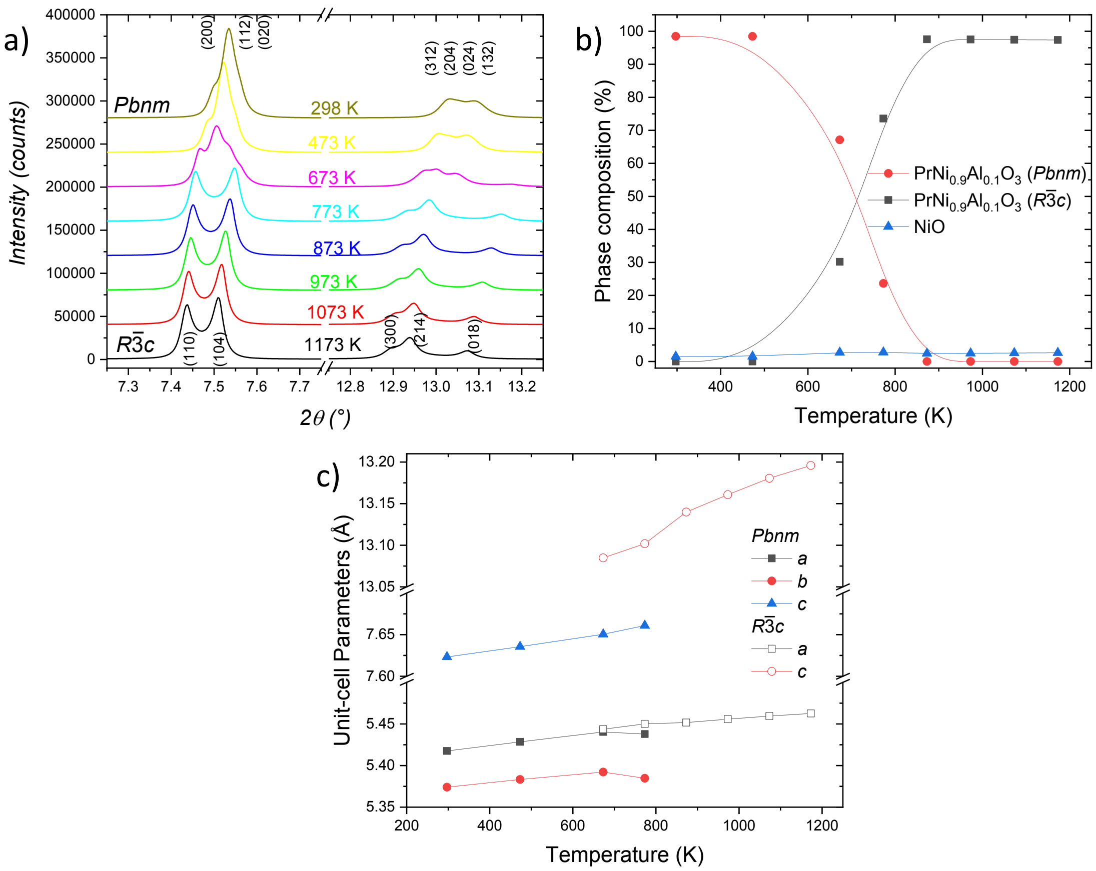

Fig. S3: Thermal evolution of the SXRD patterns for selected lines (a); phase composition (b), and unit-cell parameters (c).

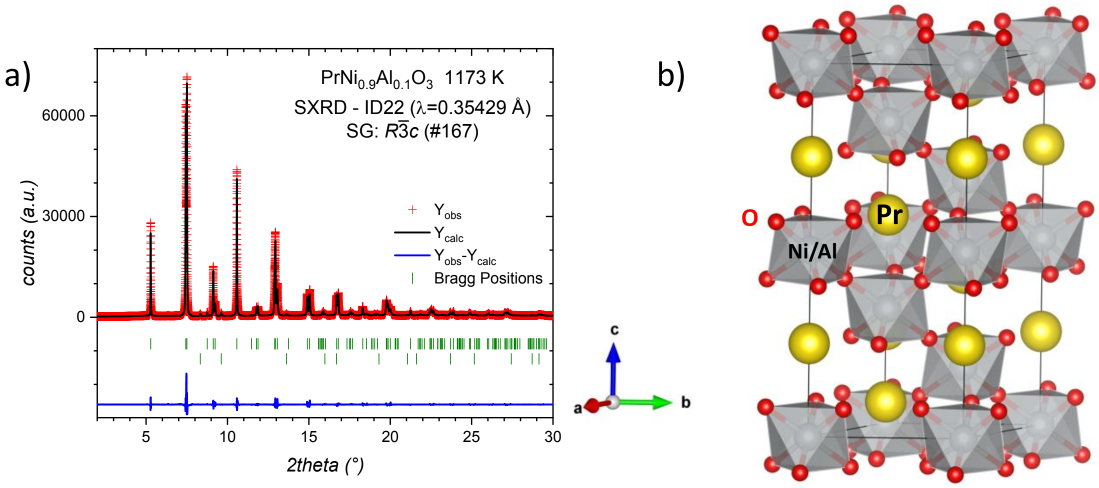

Fig. S4: (a) Rietveld refinement from SXRD data at 1173 K for $PrNi_{0.9}Al_{0.1}O_3$ in the rhombohedral space group ($R\bar{3}c$). Observed (red crosses) and calculated (black line) SXRD profile. The two series of Bragg reflections (green ticks) denote the rhombohedral phase and NiO. (b) Schematic view of the rhombohedral crystal structure of $PrNi_{0.9}Al_{0.1}O_3$ at 1173 K.

Table S2: Main crystallographic results of $PrNi_{0.9}Al_{0.1}O_3$ from SXRD data at 1173 K.

Rombohedral symmetry, space-group $R\bar{3}c$ with unit-cell parameters: a = 5.46260(3) Å, c = 13.19592(8) Å and V = 341.01(1) Å^3.

**Atom**	***X***	***y***	***z***	U_{iso} **(Å^2)**	**Occ. (<1)**
Pr	0.00000	0.00000	0.25000	0.0224(2)	
Ni	0.00000	0.00000	0.00000	0.0123(3)	0.9
Al	0.00000	0.00000	0.00000	0.0123(3)	0.1
O1	0.4489(4)	0.00000	0.25000	0.032(2)	

Impurity: NiO (2.6% w/w)
Reliability Factors: R_p = 6.42%, R_{wp} = 8.74%, R_{exp} = 1.15%, R_{Bragg} = 2.38%, χ^2 = 67.1

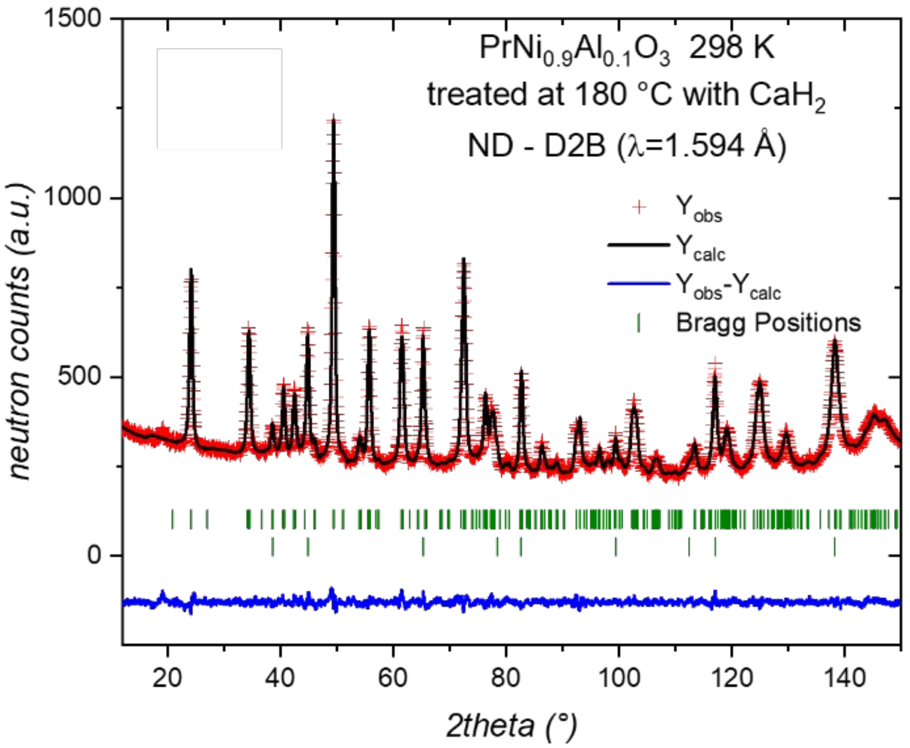

Fig. S5: Rietveld refinement from NPD data at room temperature for $PrNi_{0.9}Al_{0.1}O_3$ treated at 180 °C with CaH_2. Observed (red crosses) and calculated (black line) NPD profiles. Two series of Bragg reflections (green ticks) denote to the orthorhombic phase (*Pbnm*) and NiO.

Table S3: Main crystallographic results of $PrNi_{0.9}Al_{0.1}O_3$ treated at 180 °C with CaH_2 from NPD data at room temperature.

Orthorhombic symmetry, space-group *Pbnm* with unit-cell parameters: *a* = 5.4159(3) Å, *b* = 5.3708(3) Å, *c* = 7.6194(4) Å, and *V* = 221.63(2) Å^3.

Atom	*x*	*y*	*z*	U_{iso} (Å^2)	Occ. (<1)
Pr	0.998(2)	−0.028(1)	0.25000	0.0110(9)	
Ni	0.50000	0.00000	0.00000	0.0096(6)	0.9
Al	0.50000	0.00000	0.00000	0.0096(6)	0.1
O1	0.0673(9)	0.510(1)	0.25000	0.013(1)	0.99(1)
O2	0.7733(7)	0.2199(7)	0.0339(4)	0.017(1)	

Impurity: NiO (1.4% w/w)
Reliability Factor: R_p = 1.78%, R_{wp} = 2.28%, R_{exp} = 2.10%, R_{Bragg} = 2.04%, χ^2 = 1.18

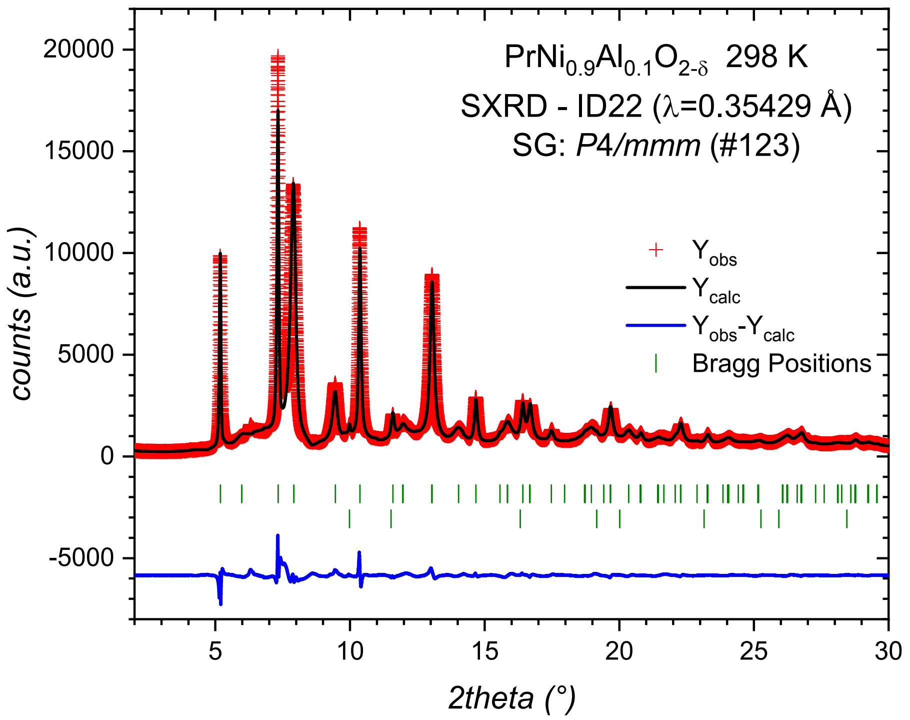

Fig. S6: (a) Rietveld refinement of the SXRD pattern at room temperature for $PrNi_{0.9}Al_{0.1}O_{2+\delta}$. Observed (red crosses) and calculated (black line) SXRD profile.

Table S4: Main crystallographic results of $PrNi_{0.9}Al_{0.1}O_{2+\delta}$ from SXRD data at room temperature.

Tetragonal symmetry, space-group *P*4/*mmm* with unit-cell parameters: *a* = 3.93009(6) Å, *c* = 3.40846(8) Å, and *V* = 52.646(2) Å^3.

**Atom**	***x***	***y***	***z***	**U_{iso} (Å^2)**	**Occ. (<1)**
Pr	0.50000	0.50000	0.50000	0.0161(2)	
Ni	0.00000	0.00000	0.00000	0.0033(2)	0.9
Al	0.00000	0.00000	0.00000	0.0033(2)	0.1
O1	0.50000	0.00000	0.00000	0.0160(7)	

Impurity: Ni^0 (1.24% w/w)
Reliability Factors: R_p = 5.37%, R_{wp} = 6.47%, R_{exp} = 0.86%, R_{Bragg} = 3.08%, χ^2 = 56.8

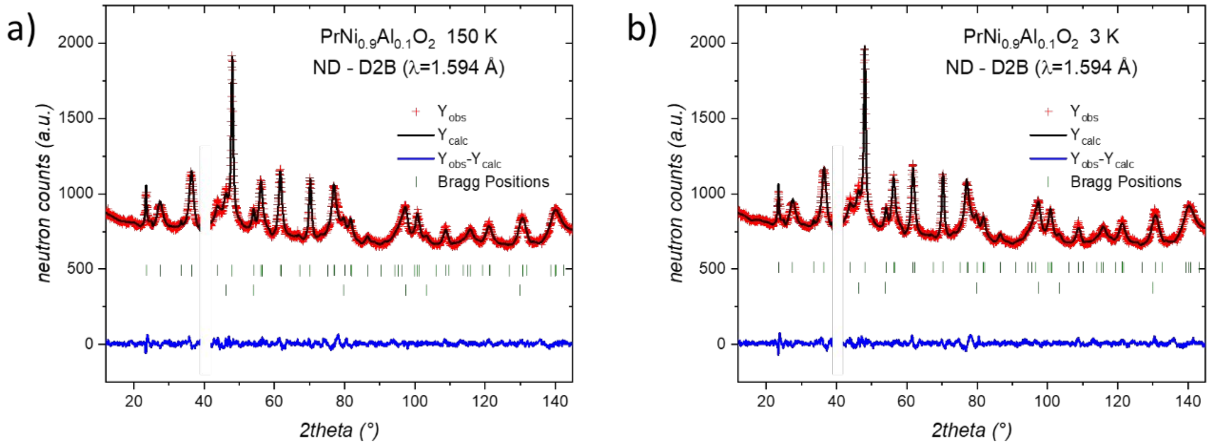

Fig. S7: Rietveld refinement from NPD data at (a) 150 and (b) 3 K.

Table S5: Main crystallographic results of $PrNi_{0.9}Al_{0.1}O_2$ from NPD data at 150 K.

System tetragonal, space-group: *P*4/*mmm* with unit-cell parameters: *a* = 3.9215(2) Å, *c* = 3.3687(4) Å, and *V* = 51.80(1) Å^3.

**Atom**	***x***	***y***	***Z***	U_{iso} **(Å^2)**	**Occ. (<1)**
Pr	0.50000	0.50000	0.50000	0.00127	
Ni	0.00000	0.00000	0.00000	0.00127	0.90000
Al	0.00000	0.00000	0.00000	0.00127	0.10000
O1	0.50000	0.00000	0.00000	0.0065(5)	
O2	0.00000	0.00000	0.50000	0.01267	0.095(9)
H	0.182(9)	0.182(9)	0.50000	0.01267	0.039(5)

Impurity: Ni (2.1% w/w)
Reliability Factor: R_p = 1.36%, R_{wp} = 1.75%, R_{exp} = 1.42%, R_{Bragg} = 0.77, χ^2 = 1.51

Table S6: Main crystallographic results of $PrNi_{0.9}Al_{0.1}O_2$ from NPD data at 3 K.

System tetragonal, space-group: *P*4/*mmm* with unit-cell parameters: *a* = 3.9199(2) Å, *c* = 3.3593(4) Å, and *V* = 51.617(7) Å^3.

**Atom**	***x***	***y***	***Z***	U_{iso} **(Å^2)**	**Occ. (<1)**
Pr	0.50000	0.50000	0.50000	0.00127	
Ni	0.00000	0.00000	0.00000	0.00127	0.90000
Al	0.00000	0.00000	0.00000	0.00127	0.10000
O1	0.50000	0.00000	0.00000	0.0044(6)	
O2	0.00000	0.00000	0.50000	0.01267	0.095(9)
H	0.174(8)	0.174(8)	0.50000	0.01267	0.042(5)

Impurity: Ni (2.0% w/w)
Reliability Factor: R_p = 1.35%, R_{wp} = 1.77%, R_{exp} = 1.42%, R_{Bragg} = 0.78, χ^2 = 1.56

Table S7: Temperature-dependent EXAFS parameter (d_j, average path distance) were refined from Ni *K*-edge spectra for $PrNi_{0.9}Al_{0.1}O_2$ infinite-layer using *Artemis* software. The reliability factor (*R*-factor) of the fitting is also provided, indicating the quality of the fit.

T (K)	d_{Ni-O} (Å)	$d_{Ni\cdots Ni}$ (Å)	$d_{Ni\cdots Pr}$ (Å)	R-factor
20	1.965(7)	2.480(5)	3.249(7)	0.0049
40	1.968(7)	2.481(6)	3.250(7)	0.0052
60	1.967(7)	2.480(6)	3.249(8)	0.0055
100	1.967(7)	2.480(5)	3.249(8)	0.0050
150	1.969(8)	2.482(7)	3.248(11)	0.0077
200	1.966(5)	2.478(5)	3.246(8)	0.0036
250	1.966(5)	2.478(4)	3.246(8)	0.0031
290	1.965(4)	2.477(4)	3.245(8)	0.0027

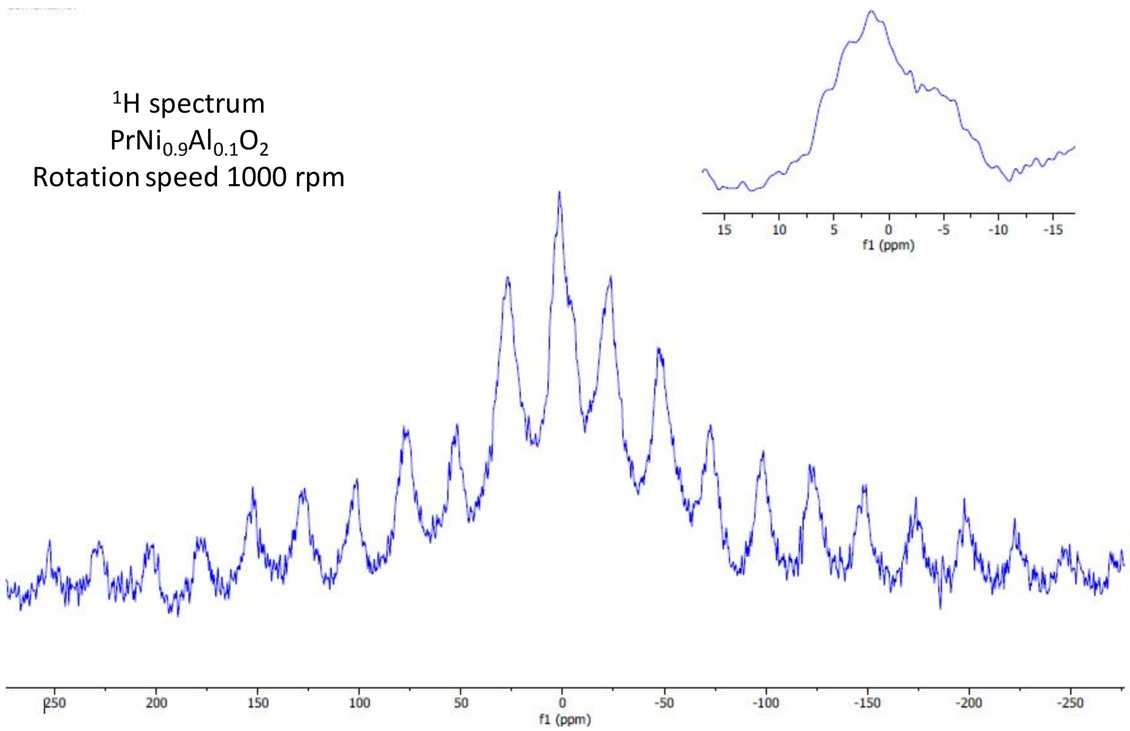

Fig. S8: NMR ^{1}H spectrum of $PrNi_{0.9}Al_{0.1}O_2$ infinite-layer phase. The H signal is strongly convoluted with the paramagnetism of Pr, indicating a strong interaction or bonding between both elements.

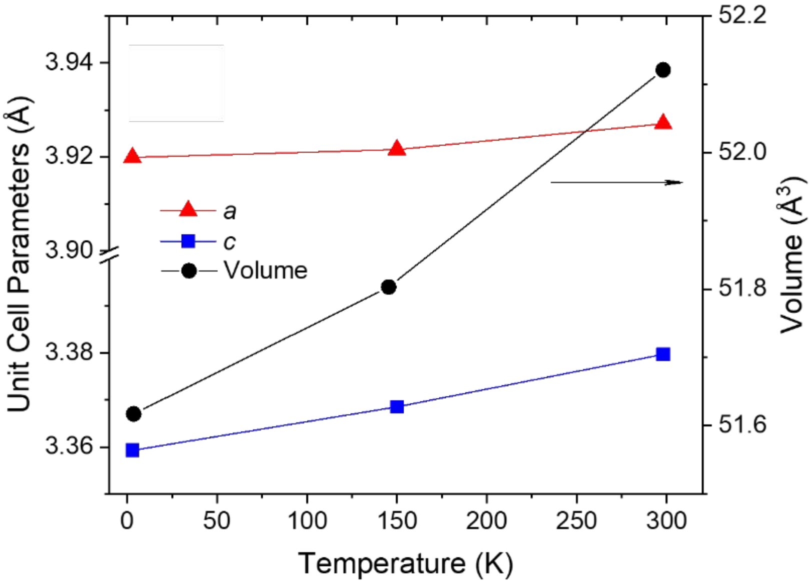

Fig. S9: Unit-cell parameters and volume versus temperature derived from Rietveld refinements of NPD data of $PrNi_{0.9}Al_{0.1}O_2$ infinite-layer phase.

References

(1) Gainza, J.; López, C. A.; Serrano-Sánchez, F.; Rodrigues, J. E. F. S.; Rosa, A. D.; Sobrados, M. I.; Nemes, N. M.; Biskup, N.; Fernández-Díaz, M. T.; Martínez, J. L.; Alonso, J. A. Evidence of Hydrogen Content and Monovalent Ni Oxidation State in Non-Superconducting Bulk Anchored Infinite-Layer Nickelates. Cell Rep Phys Sci 2023, 4 (12), 101724. https://doi.org/10.1016/j.xcrp.2023.101724.
